# Anti-Inflammatory and Antioxidant Activities of Lipophilic Fraction from *Liriope platyphylla* Seeds Using Network Pharmacology, Molecular Docking, and In Vitro Experiments

**DOI:** 10.3390/ijms241914958

**Published:** 2023-10-06

**Authors:** Van-Long Truong, Yeon-Ji Bae, Razanamanana H. G. Rarison, Ji-Hong Bang, So-Yoon Park, Woo-Sik Jeong

**Affiliations:** 1 School of Food Science & Biotechnology, College of Agriculture and Life Sciences, Kyungpook National University, Daegu 41566, Republic of Korea; truonglongpro@gmail.com (V.-L.T.); qoduswl530@naver.com (Y.-J.B.); rhanitranirina@gmail.com (R.H.G.R.); wlghd6780@naver.com (J.-H.B.); thdbs1958@naver.com (S.-Y.P.); 2 Food and Bio-Industry Research Institute, School of Food Science & Biotechnology, College of Agriculture and Life Sciences, Kyungpook National University, Daegu 41566, Republic of Korea

**Keywords:** antioxidant, anti-inflammation, *Liriope platyphylla* seed, network pharmacology, molecular docking

## Abstract

Antioxidant and anti-inflammatory mechanisms counteract the pathogenesis of chronic diseases, such as diabetes, aging, and cancer. Therefore, enhancing antioxidant and anti-inflammatory functions may help manage these pathological conditions. This study aimed to assess the antioxidant and anti-inflammatory potentials of lipophilic fraction of *Liriope platyphylla* seeds (LLPS) using network pharmacology, molecular docking, and in vitro experiments. Here GC–MS analysis tentatively identified forty-three lipophilic compounds in LLPS. LLPS exhibited powerful antioxidant activity, according to the results from chemical-based antioxidant assays on DPPH, ABTS^+^, superoxide anion, hydrogen peroxide, nitric oxide, and hydroxyl radicals scavenging, lipid peroxidation, reducing antioxidant powers, and total antioxidant capacity. Additionally, LLPS enhanced cellular antioxidant capacity by inhibiting reactive oxygen species formation and elevating antioxidant enzyme levels, including catalase and heme oxygenase-1. Moreover, LLPS attenuated inflammatory response by reducing nitric oxide secretion and downregulating the expression of inducible nitric oxide synthase, cyclooxygenase-2, and interleukin-1β in lipopolysaccharide-treated macrophages. Network pharmacology and molecular docking analyses showed that key compounds in LPPS, particularly phytosterols and fatty acid esters, exerted antioxidant and anti-inflammatory properties through regulating NFKB1, PTGS1, PTGS2, TLR4, PRKCA, PRKCD, KEAP1, NFE2L2, and NR1l2. Overall, these data suggest that LLPS may be a potential antioxidant and anti-inflammatory agent for developing functional foods.

## 1. Introduction

Systemic, or chronic, inflammation is closely associated with the pathologies of a wide variety of diseases, such as inflammatory bowel disease, psoriasis, atherosclerosis, and cancer [1]. During inflammation, a large amount of pro-inflammatory mediators and cytokines, including nitric oxide (NO), prostaglandins, inducible nitric oxide (iNOS), cyclooxygenase-2 (COX-2), interleukin (IL)-1β, and tumor necrosis factor alpha (TNF-α), are released [2]. In addition, activated inflammatory cells also excrete reactive oxygen species (ROS), reactive nitrogen species (RNS), and other oxidants at the sites of inflammation, exaggerating pathological conditions [3].

ROS and RNS, including both free radical and non-free radical species such as superoxide anion radical, hydrogen peroxide, hydroxyl radical, and nitric oxide, are the products of normal cellular metabolisms. ROS/RNS overproduction overwhelms antioxidant defense systems, leading to oxidative/nitrosative stresses that damage cellular structures and signaling pathways and ultimately trigger pathogenesis and the aging process [4,5]. Therefore, the maintenance of redox homeostasis is a vital aspect of living organisms to prevent cells from oxidative/nitrosative stresses and prevent diseases.

*Liriope platyphylla* is an herbaceous perennial seed-producing plant belonging to the Liliaceae family and is widely distributed in mountainous areas of temperate regions of East Asia. It has been used as food and traditional medicine for cough, asthma, lung inflammation, and neurodegenerative diseases in Korea, China, and Japan [6,7]. Recent studies have confirmed the biological effects of *L. platyphylla* roots, including anti-inflammatory, anti-cancer, anti-diabetic, and anti-obese activities, as well as neuroprotective and hepatoprotective effects [8,9]. Additionally, a double-blinded randomized placebo-controlled trial has shown that *L. platyphylla* extract, administered at a dosage of 1000 mg/day for four weeks, improves respiration functions in heathy volunteers without any observed adverse effects, confirming its safety and nontoxicity in use [10]. Red *L. platyphylla*, produced through a steaming process, dose not exhibit significant toxic effects in a mouse model [11]. Furthermore, its health benefits are ascribed to bioactive compounds, such as spirostanol saponins (spicatoside A and D), homoisoflavonoid, benzofurans, and sesquiterpenoids [12,13].

Compared with the root, the fruit of *L. platyphylla*, which usually ripens from October to December, has received less attention and remained underutilized. A *L. platyphylla* fruit comprises a seed and seed coat of black color containing some anthocyanins, such as delphinidin-3-*O*-glucoside, delphinidin-3-*O*-rutinoside, cyanidin-3-*O*-glucoside, petunidin-3-*O*-glucoside, petunidin-3-*O*-rutinoside, malvidin-3-*O*-glucoside, and malvidin-3-*O*-rutinoside [14]. In addition, *L. platyphylla* fruits have been found to exhibit several biological properties, such as antioxidant and anti-aging activities, and tyrosinase and collagenase inhibition [14,15]. However, to the best of our knowledge, only a few studies have examined the fruit’s phytochemical profiles and biological activities. Moreover, in terms of phytochemical compositions and biological properties, study on lipophilic fraction of *L. platyphylla* seeds has not been investigated. Therefore, this study aimed to elucidate the anti-inflammatory and antioxidant potentials of lipophilic fraction of *L. platyphylla* seeds (LLPS) using network pharmacology analysis and the experimental approaches.

## 2. Results and Discussion

### 2.1. Chemical Composition of LLPS

*L. platyphylla* is well known as a steroidal saponin-rich plant that reveals various health benefits due to its bioactive components, such as spirostanol saponins and homoisoflavonoids [14]. However, the chemical compositions in *L. platyphylla* seeds, especially lipophilic compounds, have not been investigated. This study for the first time reported the chemical components of lipophilic fraction of *L. platyphylla* seeds. By GC–MS analysis, 43 compounds, forming 90.76% of total lipophilic compounds, were tentatively identified in LLPS, as listed in Table 1. These compounds could be classified into six groups, including fatty acids and esters (63.73%), hydrocarbons (3.02%), phytosterols (11.78%), terpenes (3.09%), tocols (1.87%), and others (7.18%). LLPS contains unsaturated fatty acids, such as linoleic acid and oleic acid, which exert various health-promoting effects [16,17]. Considerable amounts of esters such as glyceryl monooleate, ethyl linoleate, 9-octadecenoic acid ethyl ester, glycidyl oleate, 2-palmitoylglycerol, and ethyl palmitate were detected in the LLPS. Notably, LLPS is a rich source of phytosterols, containing approximately 120 mg of phytosterols per g LLPS. Among them, clionasterol emerges as the predominant phytosterol, followed by fucosterol, stigmasterol, and cycloartenol. Stigmasterol has been found in the roots of *L. platyphylla* and other *Liriope* species [18]. These phytosterols exert a broad spectrum of biological properties, such as antioxidant, anti-inflammation, chemopreventive and anti-atherosclerotic effects, as well as management of obesity and metabolic disorders [19,20]. LLPS also contains α-tocopherol, which is the most active isoform of vitamin E and importantly contributes to cellular antioxidant defense, cellular membrane protection, and disease prevention [21,22]. Some terpenes found in the LLPS, such as squalene, 2,3-oxidosqualene, and α-selinene, are acknowledged to enhance human health due to their antioxidant and anti-inflammatory properties [23,24]. In addition, LLPS contains hydrocarbons and other compounds, although their biological significance remains unknown. Overall, LLPS may be a useful functional component since it contains a variety of bioactive compounds that bring about various health-promoting effects.

### 2.2. Antioxidant and Anti-Inflammatory Mechanisms of LLPS Based on Network Pharmacology Analysis

#### 2.2.1. Network Construction and Analysis

**Figure 1:**
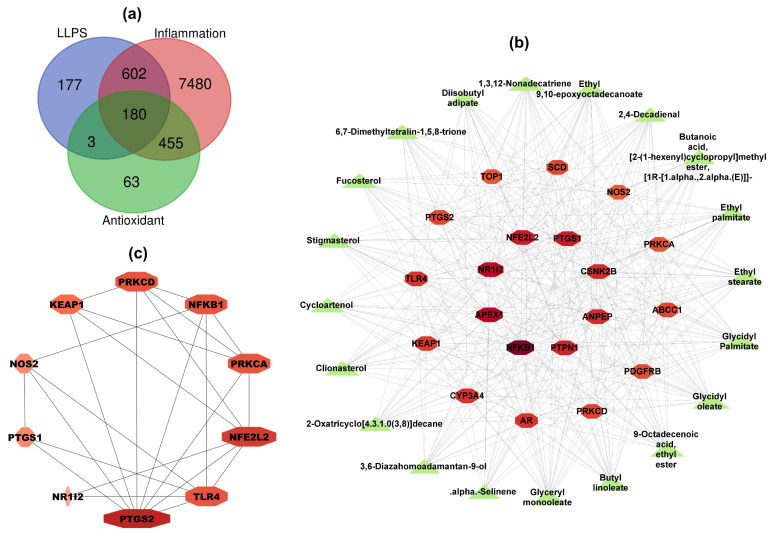
Network pharmacology analysis for antioxidant and anti-inflammatory mechanisms of LLPS. (**a**) Venn diagram of LLPS compounds, inflammation, and antioxidant targets. (**b**) Compound–target network. The green triangle nodes represent the active compounds of LLPS. The red-shade octagonal nodes represent the inflammation and antioxidant target genes of LLPS. (**c**) Protein–protein interaction (PPI) network of the top 10 core target genes evaluated by the MCC method. The color-shade nodes represent the degree of binding between proteins. A darker node represents a higher degree, and a lighter indicates a lower degree. LLPS: Lipophilic fraction from *L. platyphylla* seeds.

A total of 962 target genes of 43 LLPS compounds were collected from the Swiss Target Prediction and SuperPred databases. A total of 9418 target genes, including 8717 genes related to inflammation and 701 genes related to antioxidant, were obtained from the GeneCards, OMIM, and CTD databases. After overlapping, a Venn diagram revealed 180 intersection target genes among inflammation, antioxidant, and bioactive compound targets (Figure 1a).

To elucidate the relationship between bioactive compounds in LLPS and intersection target genes, a compound–target network was created using Cytoscape 3.9.1 software (Figure S1). The network revealed 223 nodes (43 compound nodes and 180 target nodes) and 2156 edges, suggesting that one compound might influence multiple target genes, and different compounds might share the same target genes [25]. The average degree of the 43 active compounds in the network was 50.13. The top 20 active compounds with the highest degree values included glycidyl palmitate, glycidyl oleate, 1,3,12-nonadecatriene, ethyl stearate, ethyl 9,10-epoxyoctadecanoate, fucosterol, cycloartenol, clionasterol, 9-octadecenoic acid ethyl ester, butyl linoleate, stigmasterol, 2-oxatricyclo[4.3.1.0(3,8)]decane, glyceryl monooleate, 6,7-dimethyltetralin-1,5,8-trione, diisobutyl adipate, [1R-[1.alpha.,2.alpha.(E)]]-butanoic acid [2-(1-hexenyl)cyclopropyl]methyl ester, alpha-selinene, ethyl palmitate, 3,6-diazahomoadamantan-9-ol, 2,4-decadienal. These compounds, mainly phytosterols and fatty acid esters, are likely bioactive components contributing to antioxidant and anti-inflammatory properties of LLPS. Figure 1b illustrates the compound–target network of the top 20 active compounds and top 20 core target genes.

To understand potential antioxidant and anti-inflammatory mechanisms of LLPS, the top 20 target genes from the compound–target network were introduced to the STRING database to determine the protein–protein interaction (PPI). The PPI network was built using Cytoscape software and further analyzed by the maximal clique centrality (MCC) method in CytoHubba to depict the core targets. As shown in Figure 1c, the top 10 Hub genes were identified, including PTGS2, TLR4, NFE2L2, PRKCA, NFKB1, PRKCD, KEAP1, NOS2, PTGS1, and NR1l2; among them, PTGS2 interacted the most with other targets.

Toll-like receptor 4 (TLR4), a transmembrane pattern recognition receptor, plays a pivotal role in immune responses and can be activated by pathogen-associated molecular patterns, such as endotoxins (e.g., lipopolysaccharide). TLR4 activation triggers intracellular signaling pathways, such as nuclear factor-kappa B (NFKB), leading to the expression of multiple genes like prostaglandin-endoperoxide synthase 2 (PTGS2), inducible nitric oxide synthase 2 (NOS2), and interleukins, which provoke local or systemic inflammation [26,27]. PTGS1 and PTGS2, also known as COX-1 and COX-2, respectively, catalyze the conversion of arachidonic acid into prostaglandins, influencing both human physiology and pathology [28]. Particularly, COX-2 is rapidly upregulated in response to diverse inflammatory stimuli and oxidative stress, and its dysregulation is closely linked to pathological conditions, such as inflammation and cancer. Inhibiting COX-2 has shown promise in managing inflammation-related diseases and reducing cancer incidence and prevalence [29,30]. Additionally, NOS2, or iNOS, is a rate-limiting enzyme that synthesizes NO, a pro-inflammatory mediator of the immunoinflammatory process implicated in the development of several chronic diseases [31].

Kelch-[ECH]-associated protein 1 (KEAP1) is a negative regulator of nuclear factor erythroid 2-related factor 2 (NFE2L2, or Nrf2), which is a master transcription factor mediating redox homeostasis. Under physiological condition, NFE2L2 is sequestered in the cytosol by KEAP1. However, in response to electrophiles and oxidative stress, NFE2L2-KEAP1 complex is dissociated, leading to NFE2L2 liberation and subsequent nuclear translocation to drive the transcription of a number of antioxidant genes, namely, heme oxygenase 1 (HO-1), catalase (CAT), glutathione peroxidase (GPx), and superoxide dismutase (SOD) [32].

Nuclear receptor subfamily 1 group I member 2 (NR1I2), also known as pregnane X receptor (PXR), is a nuclear receptor involved in xenobiotic metabolism and the regulation of inflammatory response by suppressing the NF-κB signaling pathway [33]. Moreover, PXR has been shown to possess a protective role against oxidative stress by regulating phase I and II enzymes, such as cytochrome P450s and glutathione *S*-transferase [34]. Additionally, protein kinase C alpha (PRKCA) and protein kinase C delta (PRKCD), belonging to the serine/threonine-specific protein kinase C family, participate in multiple signaling transduction pathways associated with cell proliferation, apoptosis, angiogenesis, and immune response [35]. PRKCA has been implicated in mediating LPS-stimulated iNOS and IL-1α expression in RAW 264.7 macrophages [36], whereas RAW 264.7 cells overexpressing dominant-negative mutant of PRKCA showed reduced COX-2 expression in response to LPS stimulation [37]. Furthermore, PRKCD is involved in Nrf2 activation via p62 phosphorylation [38]. Taken together, these findings suggest that LLPS may exert antioxidant and anti-inflammatory properties through regulating NFKB1, PTGS1, PTGS2, TLR4, PRKCA, PRKCD, KEAP1, NFE2L2, and NR1l2.

#### 2.2.2. Gene Ontology (GO) and Kyoto Encyclopedia of Genes and Genomes (KEGG) Pathway Enrichment Analyses

**Figure 2:**
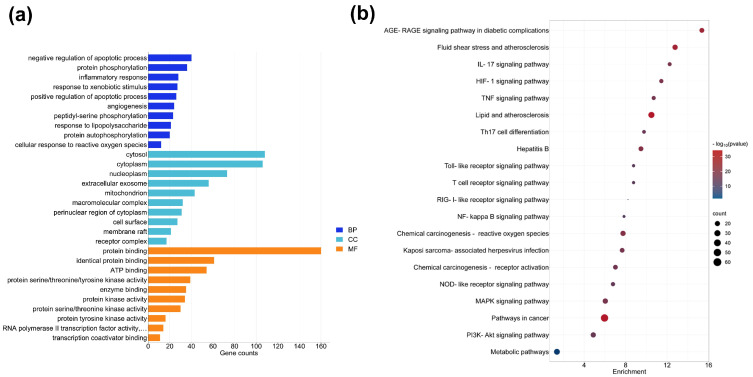
GO and KEGG pathway enrichment analyses. (**a**) The bar chart represents the top 10 enriched GO terms (*p* < 0.05) in the biological processes (BP), cellular components (CC), and molecular functions (MF) categories. (**b**) The bubble chart displays the top 20 enriched KEGG pathways.

To further explore the underlying anti-inflammatory and antioxidative mechanisms of LLPS, GO and KEGG enrichment analyses were accomplished using the DAVID bioinformatic resources. The GO analysis identified a total of 867 GO items (*p* ≤ 0.05), comprising 629 biological processes (BP), 76 cell components (CC), and 162 molecular functions (MF) entries. The top 10 enriched entries in the BP, CC, and MF categories are displayed in Figure 2a. The results showed that BP was mainly associated with negative/positive regulation of the apoptotic process, protein phosphorylation, inflammatory response, response to xenobiotic stimuli/lipopolysaccharide, and cellular response to reactive oxygen species. The primarily CC terms included cytosol, cytoplasm, nucleoplasm, mitochondria, and membrane raft, while the main MF terms were protein binding, ATP binding, and protein kinase activity. Additionally, the KEGG pathway analysis indicated that common targets of bioactive compounds in LLPS were primarily enriched in the AGE–RAGE signaling pathway in diabetic complications, fluid shear and atherosclerosis, IL-17 signaling pathway, TNF signaling pathway, HIF-1 signaling pathway, toll-like receptor (TLR) signaling pathway, NF-kappa B signaling pathway, chemical carcinogenesis-reactive oxygen species, NOD-like receptor (NLR) signaling pathway, MAPK signaling pathway, and PI3K-Akt signaling pathway (Figure 2b).

The NF-κB signaling pathway is involved in multiple aspects of innate and adaptive immune systems. As a key regulator of inflammatory response, NF-κB governs the production of various pro-inflammatory mediators, cytokines, chemokines, and growth factors, contributing to the pathogenic processes of inflammatory diseases. In addition, NF-κB takes part in the activation and differentiation of inflammatory T cells and other immune cells as well as the regulation of inflammasome [27]. Upon exposure to diverse stimuli, multiple upstream signaling pathways, such as the TLR, TNF, IL-17, NLR, MAPK, and PI3K-Akt signaling pathways, converge on NF-κB to produce adaptive responses. For instance, the TLR signaling pathway, activated by various pathogen-associated molecular patterns, plays a crucial role in inflammation by inducing the key transcriptional regulator NF-κB. Once activated, NF-κB triggers the generation of pro-inflammatory cytokines like pro-IL-1β and pro-IL-18, which are subsequently cleaved by NLRP3 inflammasome, a well-studied NLR signaling pathway, to secrete mature IL-1β and IL-18 [27]. Additionally, the TNF and IL-17 signaling pathways regulate a wide spectrum of cellular events such as proliferation, apoptosis, stress response, and inflammation. Consequently, these pathways are intimately associated with the pathogenesis of inflammatory disorders, including inflammatory bowel diseases, rheumatoid arthritis, autoimmune diseases, and cancer [39,40].

The HIF-1 signaling pathway plays a crucial role in regulating genes related to inflammation, metabolisms, and cell survival. Cross talk between HIF-1α and NF-κB is essential for inflammatory functions by driving the expression of cytokines [25]. In response to hypoxia, HIF can mitigate the formation of ROS and oxidative stress by inducing the expression of Nrf2-mediated HO-1 enzymes [41]. Furthermore, accumulating evidence suggests a reciprocal relationship between ROS, Nrf2 signaling, and HIF-1α stabilization and transactivation. Nrf2 knockout or ROS elimination attenuates the activation of the HIF-1 signaling pathway [42]. Additionally, intracellular signal transduction pathways, such as PI3K-Akt and MAPK cascades, participate in various biological processes, including proliferation, apoptosis, and inflammatory response, through regulating downstream transcription factors. The PI3K-Akt signaling pathway contributes to the secretion of pro-inflammatory cytokines via activating the NF-κB pathway and is implicated in the pathogenesis of inflammatory disorders [43]. Similarly, the MAPK signaling pathway is responsible for transmitting extracellular signals to intracellular responses, encompassing proliferation, apoptosis, stress response, and inflammation. Moreover, MAPK cascades have been found to mediate both inflammatory response by regulating the NF-κB pathway and antioxidant defense by activating Nrf2/phase II enzymes [44,45].

Overall, most of the enriched pathways are associated with inflammation and oxidative stress, suggesting that LLPS may exert anti-inflammatory and antioxidant activities by modulating these pathways.

### 2.3. Molecular Docking Verification

**Figure 3:**
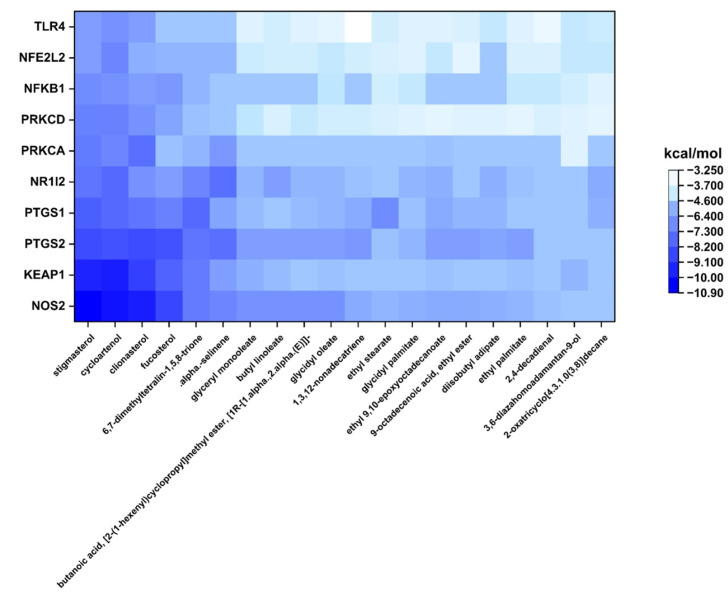
Heatmap represents the binding energies (kcal/mol) of the top 20 bioactive compounds in LLPS with the top 10 core target proteins.

**Figure 4:**
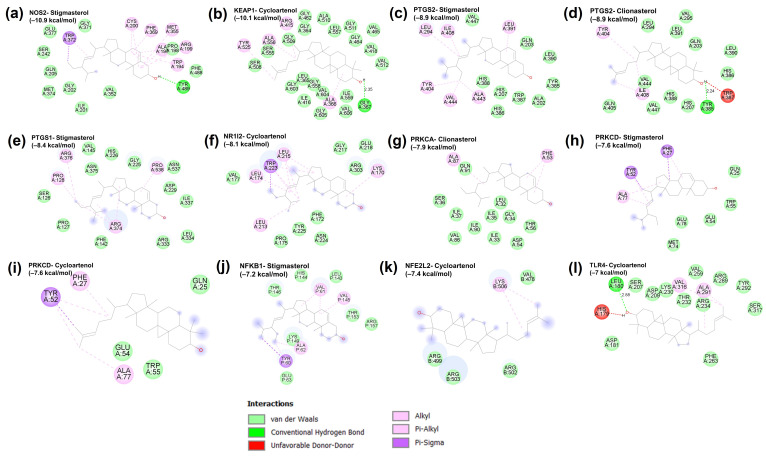
Representative molecular docking results of bioactive ingredients in LLPS and the hub genes. (**a**) NOS2–Stigmasterol (score = −10.9); (**b**) KEAP1–Cycloartenol (score = −8.1); (**c**) PTGS2–Stigmasterol (score = −8.9); (**d**) PTGS2–Clionasterol (score = −8.9); (**e**) PTGS1–Stigmasterol (score = −8.4); (**f**) NR1l2–Cycloartenol (score = −8.1); (**g**) PRKCA–Clionasterol (score = −7.9); (**h**) PRKCD–Stigmasterol (score = −7.6); (**i**) PRKCD–Cycloartenol (score = −7.6); (**j**) NFKB1–Stigmasterol (score = −7.2); (**k**) NFE2L2–Cycloartenol (score  =  −7.4); (**l**) TLR4–Cycloartenol (score  =  −7.0).

The binding potentials between the top 10 Hub genes and the 20 active compounds of LLPS were assessed using molecular docking simulation. The results of molecular docking analysis are summarized in Table S1. Binding energy serves as an indicator for assessing the interaction strength between a compound and a protein target, with lower binding energies indicating more stable and stronger binding interactions. Generally, an energy value below −5.0 kcal/mol shows a favorable binding conformation between the compound and protein [46]. The heatmap illustrates that 146 out of the 200 docking results had binding energies below −5.0 kcal/mol, and 42 results had binding energies below −7.0 kcal/mol (Figure 3). The binding poses of representative docking results are shown in Figure 4.

Considered as a strong interaction with binding energy below −7.0 kcal/mol, cycloartenol exhibited strong binding interactions with all 10 core targets, including NOS2 (−10.4 kcal/mol), KEAP1 (−10.1 kcal/mol), PTGS2 (−8.7 kcal/mol), PTGS1 (−8.2 kcal/mol), NR1I2 (−8.1 kcal/mol), PRKCA (−7.3 kcal/mol), PRKCD (−7.6 kcal/mol), NFKB1 (−7.1 kcal/mol), NFE2L2 (−7.4 kcal/mol), and TLR4 (−7.0 kcal/mol). Cycloartenol, a precursor of numerous sterols, has been demonstrated to exert antioxidant, anti-inflammatory, anti-tumor, and antibiotic activities [47,48]. However, its precise mechanism of action remains elusive. Previous research has highlighted the protective ability of cycloartenol against benzoyl peroxide and ultraviolet-B-caused oxidative stress and skin carcinogenesis by augmenting the activities of CAT, GPx, glutathione reductase, glucose-6-phosphate dehydrogenase, quinone reductase, and glutathione-S-transferase in mouse skins [49]. Stigmasterol had robust binding activities with NOS2 (−10.9 kcal/mol), KEAP1 (−9.9 kcal/mol), PTGS2 (−8.9 kcal/mol), PTGS1 (−8.4 kcal/mol), NR1I2 (−7.8 kcal/mol), PRKCA (−7.7 kcal/mol), PRKCD (−7.6 kcal/mol), and (−7.1 kcal/mol). Stigmasterol, a widely distributed phytosterol, possesses a range of pharmacological effects, including antioxidant, anti-inflammatory, anti-bacterial, anti-osteoarthritic, and anti-cancer properties [50,51]. It has been deciphered to reduce the levels of pro-inflammatory cytokines and mediators via the downregulation of the NF-κB pathway in various in vitro and in vivo models [50,52]. Additionally, stigmasterol exerts a neuroprotective effect against oxidative damage through enhancing the activity of GPx and SOD enzymes and activating the Keap1/Nrf2/HO-1 signaling pathway [52]. Clionasterol had stable bindings with NOS2 (−10.1 kcal/mol), KEAP1 (−9.2 kcal/mol), PTGS2 (−8.9 kcal/mol), PTGS1 (−7.8 kcal/mol), NR1I2 (−7.1 kcal/mol), PRKCA (−7.9 kcal/mol), and PRKCD (−7.0 kcal/mol). Clionasterol has been proved to possess antioxidant and anti-inflammatory abilities by inhibiting ROS generation, lipid peroxidation, and NO production, and thereby preventing particulate matter (PM)-induced skin damage [53]. The strong bindings of fucosterol with NOS2 (−9.1 kcal/mol), PTGS2 (−8.7 kcal/mol), and PTGS1 (−7.6 kcal/mol) could be associated with its anti-inflammatory activity. Previous studies indicated that fucosterol inhibited the expressions of iNOS, COX-2, IL-6, and TNF-α in LPS-treated RAW 264.7 cells through suppressing the NF-κB/p38 signaling pathway [54]. In addition, a robust binding between fucosterol and KEAP1 (−8.3 kcal/mol) supported a previous observation that fucosterol enhanced the level of Nrf2 along with antioxidant enzymes, such as CAT, SOD, and HO-1 in PM-stimulated A549 cells [55].

Alpha-selinene and 6,7-dimethyltetralin-1,5,8-trione also exhibited strong affinities with NOS2 (−7.3 and −7.7 kcal/mol, respectively), NR1I2 (−7.9 and −7.3 kcal/mol, respectively), PTGS2 (−8.0 and −7.8 kcal/mol, respectively), PTGS1 (−6.6 and −8.1 kcal/mol, respectively), and KEAP1 (−6.8 and −7.7 kcal/mol, respectively). Other compounds, including glyceryl monooleate, butyl linoleate, glycidyl oleate, 1,3,12-nonadecatriene, and 9-octadecenoic acid ethyl ester, exhibited binding energies below −7.0 kcal/mol with core target proteins. Although their biological properties have not been fully understood, some have been linked to exerting antioxidant and anti-inflammatory activities. For instance, selinene exhibits chemopreventive effects against preneoplastic lesions by increasing the activities of phase II detoxification enzymes [56]. Additionally, 9-octadecenoic acid ethyl ester inhibits the release of pro-inflammatory cytokines and mediators by downregulating the NF-κB and MAPK signaling pathways in LPS-treated RAW 264.7 macrophages [57].

Overall, these results suggest that bioactive compounds, mainly phytosterols and fatty acid esters, contribute to the antioxidant and anti-inflammatory capacities of LLPS through regulating the core target genes such as NOS2, PTGS2, PTGS1, NFKB1, and KEAP1.

### 2.4. In Vitro Antioxidant Activities of LLPS

**Figure 5:**
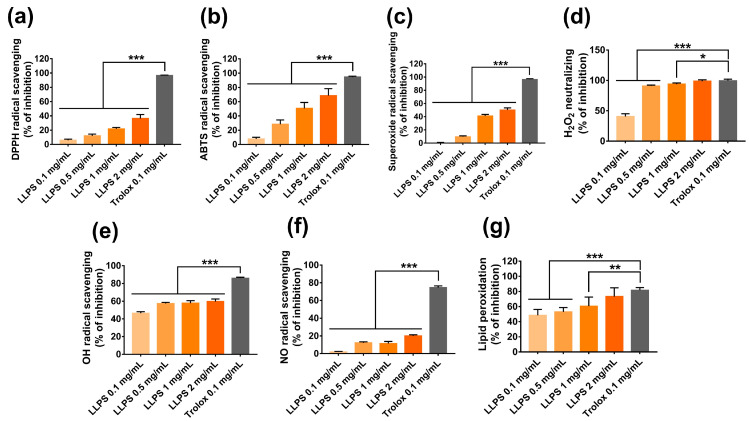
Antioxidant activity of LLPS in cell-free systems. (**a**) DPPH, (**b**) ABTS^+^, (**c**) superoxide anion radical, (**d**) H_2_O_2_, (**e**) Hydroxyl radical, (**f**) NO radical scavenging activities, and (**g**) lipid peroxidation inhibition. Results are presented as the mean ± SD. Values of * *p* < 0.05, ** *p* < 0.01, and *** *p* < 0.001 are considered statistically significant differences. LLPS: Lipophilic fraction from *L. platyphylla* seeds.

The DPPH and ABTS^+^ radical scavenging activity assays are the most common methods used to examine the antioxidant activity of extracts and phytochemicals through electron transfer and hydrogen atom transfer mechanisms [58]. As shown in Figure 5a, LLPS exhibited dose-dependent DPPH radical scavenging activity, with the percent inhibition ranging from 5.89 ± 1.17% to 36.26 ± 3.21% at the concentration range from 0.1 mg/mL to 2 mg/mL. In addition, the ABTS^+^ radical scavenging ability of LLPS increased as its concentration increased (Figure 5b). At 2 mg/mL, LLPS eliminated approximately 68.51 ± 9.77% of ABTS^+^ radicals. In general, LLPS exhibits good DPPH and ABTS^+^ radical scavenging activities, suggesting that its bioactive components possess an excellent scavenging activity.

LLPS also exhibited a concentration-dependent superoxide radical scavenging activity, with percent inhibition ranging from 8.19 ± 2.22% to 65.90 ± 3.25% at the concentration from 0.1 mg/mL to 2 mg/mL (Figure 5c). In living organisms, superoxide radical is one of the most frequently generated ROS in the mitochondria. It serves as the primary oxidant that contributes to the generation of other oxidants, namely, hydrogen peroxide, hydroxyl radical, and peroxynitrite [44]. Superoxide radicals trigger lipid peroxidation and the aging process [59].

In this study, LLPS exhibited excellent H_2_O_2_ scavenging activity at a concentration range from 0.1 mg/mL to 2 mg/mL (Figure 5d). At 2 mg/mL, LLPS eliminated approximately 98.6 ± 0.51% of H_2_O_2_, comparable to the activity of Trolox, a positive control. In addition, LLPS showed a moderate hydroxyl radical scavenging ability varying from 46.39 ± 0.46% to 59.57 ± 2.875 at the concentration range from 0.1 mg/mL to 2 mg/mL (Figure 5e). Hydrogen peroxide is a weak oxidant; however, it can interact with transition metal ions to generate highly reactive hydroxyl radicals [4]. In contrast, although having the shortest half-life among ROS, hydroxyl radical is a highly reactive oxidant that easily damages adjacent biomolecules, such as lipids, proteins, and DNA, initiating pathological conditions [59].

Nitric oxide is an abundant reactive molecule implicated in physiological and pathological events. For example, during an oxidative burst by an inflammatory response, nitric oxide may react with superoxide anions to generate peroxynitrite anion, a much more reactive oxidant that can cause lipid oxidation and DNA damage. In addition, the overproduction of nitric oxide radicals leads to a deleterious process called nitrosative stress [45]. Here, a weak nitric oxide scavenging activity was observed in the LLPS, with the percent inhibition of 19.92 ± 2.35% at the concentration of 2 mg/mL (Figure 5f).

Notably, LLPS exhibited a notable inhibition of lipid peroxidation at 0.1 mg/mL to 2 mg/mL (Figure 5g). At 2 mg/mL LLPS inhibited approximately 74% lipid peroxidation, comparable to the activity of Trolox. Lipids, especially unsaturated fatty acids, are susceptible to oxidation, giving rise to lipid peroxidation. Primary lipid peroxidation generates hydroperoxides, which cause a variety of secondary reactions that finally produce aldehydes, ketones, and other substances [45]. Lipid oxidation is a major cause of off-flavor and loss of nutrients in foods [60]. In living organisms, lipid peroxidation by reactive species can cause damage to the cellular membrane, enhancing the risk of pathological conditions [61].

The TAC was assessed based on the reduction of Mo (IV) to Mo (V) by antioxidant compounds. In the present study, LLPS showed a potent antioxidant ability, with a TAC value of 104.55 ± 4.69 mg TE/g (Table 2). The FRAP and PFRAP methods are utilized to evaluate the reducing potential of plant extracts and isolated compounds, which are based on the ability of an antioxidant to donate an electron [62]. This study showed that LLPS possessed high reducing powers, with values of 40.71 ± 1.28 mg TE/g for FRAP and 25.55 ± 0.94 mg TE/g for PFRAP (Table 2).

Overall, these findings suggest that LLPS possesses a potent antioxidant capacity, which may be ascribed to the presence of bioactive compounds that act as electron/hydrogen atom donors to scavenge free radicals and terminate the radical chain reaction.

### 2.5. Antioxidant Activity of LLPS in LPS-Stimulated RAW 264.7 Cells

**Figure 6:**
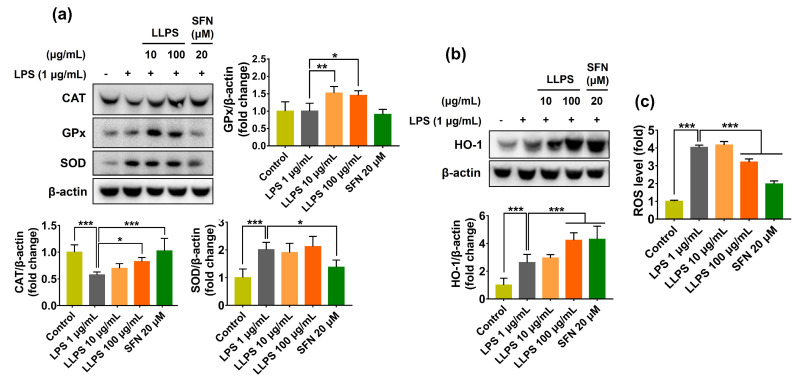
Antioxidant activity of LLPS in LPS-stimulated RAW 264.7 cells. The cells were pretreated with LLPS for 1 h prior to LPS (1 µg/mL) for an additional 6 h or 12 h. (**a**) Protein expressions of CAT, GPx, and SOD. (**b**) Protein expression of HO-1. (**c**) Intracellular ROS formation at 6 h. Results are presented as the mean ± SD. Values of * *p* < 0.05, ** *p* < 0.01, and *** *p* < 0.001 are considered statistically significant differences. LLPS: Lipophilic fraction from *L. platyphylla* seeds; SFN: Sulforaphane; LPS: Lipopolysaccharide.

The antioxidant ability of LLPS was further examined in LPS-stimulated RAW 264.7 cells. Results showed that LPS markedly decreased the level of CAT in macrophages, whereas LLPS pretreatment significantly restored the CAT level (Figure 6a). Like CAT expression, the LLPS considerably increased the GPx expression in LPS-challenged RAW 264.7 cells; particularly, the LLPS appeared more effective than the sulforaphane (SFN, a positive control). Unlike CAT and GPx levels, SOD expression was not affected by this compound in macrophages. Cellular antioxidant capacity can be achieved by directly eliminating ROS/RNS or indirectly enhancing the antioxidant defense system, such as antioxidant enzymes, or both. Endogenous antioxidant enzymes consisting of CAT, GPx, and SOD effectively act as the “ultimate antioxidants” to protect cells against a substantial oxidative attack by catalyzing various biological reactions to neutralize ROS or free radicals [63]. However, oxidative stress overwhelms the capacity of an inherent cellular antioxidant system, causing oxidative damage to the cells. Therefore, LLPS-induced levels of primary antioxidant enzymes contribute to maintaining cellular redox homeostasis.

Furthermore, LLPS pretreatment strongly elevated the HO-1 level in LPS-treated RAW 264.7 macrophages, and its efficacy was comparable to that of SFN, a well-known inducer of phase II enzymes (Figure 6b). As a result, LPS-induced ROS accumulation was significantly attenuated in the presence of LLPS (Figure 6c). Besides the primary antioxidant enzymes, phase II antioxidant enzymes, including HO-1, are also vital components of the endogenous defense system involved in neutralizing and eliminating ROS/RNS, xenobiotics, and noxious toxicants before they can damage cellular biomolecules [4].

Overall, these results suggest that LLPS possesses a potent antioxidant capacity through directly scavenging oxidants and indirectly inducing antioxidant defense systems.

### 2.6. Anti-Inflammatory Activity of LLPS in LPS-Stimulated RAW 264.7 Cells

**Figure 7:**
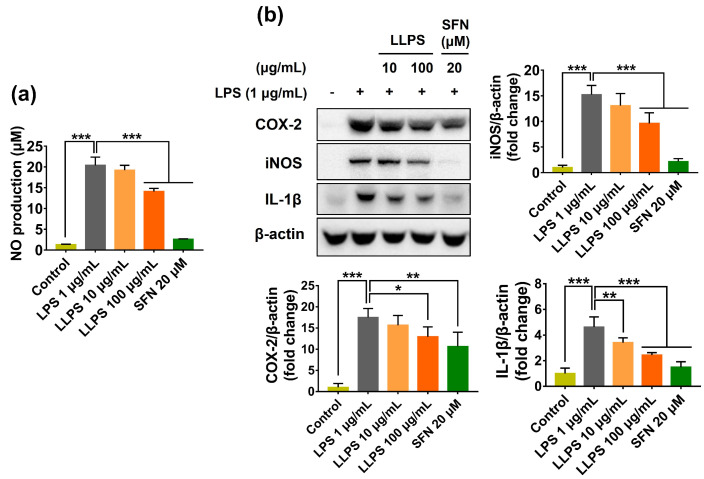
Anti-inflammatory activity of LLPS in LPS-stimulated RAW 264.7 cells. The cells were pretreated with LLPS for 1 h prior to LPS (1 µg/mL) for an additional 12 h. (**a**) NO production. (**b**) Protein expressions of COX-2, iNOS, and IL-1β. Results are presented as the mean ± SD. Values of * *p* < 0.05, ** *p* < 0.01, and *** *p* < 0.001 are considered statistically significant differences. LLPS: Lipophilic fraction from *L. platyphylla* seeds; SFN: Sulforaphane; LPS: Lipopolysaccharide.

Next, this study evaluated the anti-inflammatory properties of lipophilic compounds from *L. platyphylla* seeds using LPS-treated RAW 264.7 macrophages. LPS (1 µg/mL) substantially elevated the NO level in the macrophage culture medium; however, LLPS pretreatment significantly decreased the NO level (Figure 7a). To further clarify the anti-inflammatory potential of LLPS, the expression levels of iNOS, COX-2, and IL-1β were examined in LPS-treated RAW 264.7 cells. LPS treatment considerably upregulated iNOS and COX-2 expression, compared with the normal control. However, LLPS significantly reduced the expression of these enzymes. Furthermore, LPS treatment markedly increased the level of IL-1β in the LPS-challenged macrophages; this effect was significantly inhibited by LLPS pretreatment (Figure 7b).

Uncontrolled inflammation can cause tissue damage; therefore, it is associated with the initiation and progression of multiple diseases, such as degenerative diseases, inflammatory bowel diseases, and cancers [1]. Macrophages are an important component of the host immune system against infection by pathogens; they are activated by different stimuli, including bacterial LPS [64]. In response to pro-inflammatory stimuli, such as endotoxins (e.g., LPS), inflammatory cytokines (e.g., tumor necrosis factor-α), and ROS/RNS, macrophages produce a broad spectrum of pro-inflammatory factors, such as NO and prostaglandins, iNOS, COX-2, and IL-1β [65]. COX-2 is an inducible enzyme responsible for producing large amounts of prostaglandins at the inflammatory site. Meanwhile, the high production of NO by iNOS causes oxidative/nitrosative stresses, triggering deleterious consequences, such as septic shock and multiple inflammatory diseases [66,67]. Based on these observations, the regulation of inflammatory responses via the inhibition of pro-inflammatory mediators and cytokines is thought to be an effective procedure for preventing inflammatory conditions.

Overall, these findings suggest that LLPS could be an effective anti-inflammatory agent via decreasing the levels of pro-inflammatory factors.

## 3. Materials and Methods

### 3.1. Materials

Lipopolysaccharide (LPS, *Escherichia coli* O127:B18), 2,2-diphenyl-1-picrylhydrazyl (DPPH), 2,2′-Azino-bis(3-ethylbenzothiazoline-6-sulfonic acid) (ABTS), sodium nitroprusside (SNP), Trolox, nitro blue tetrazolium (NBT), thiobarbituric acid (TBA), Dichlorodihydrofluorescein diacetate (DCFH-DA), and phenazine methosulfate (PMS) were obtained from Sigma (St. Louis, MO, USA). Anti-catalase (CAT), iNOS, COX-2, and peroxidase-conjugated secondary anti-rabbit antibodies were provided from Cell Signaling Technology (Beverly, MA, USA). Anti-IL-1β, glutathione peroxidase (GPx), superoxide dismutase-2 (SOD-2), and peroxidase-conjugated secondary anti-mouse antibodies were obtained from Santa Cruz Biotechnology (Santa Cruz, CA, USA). Anti-heme oxygenase-1 (HO-1) antibody was acquired from Abcam (Cambridge, UK). All other reagents were of the highest grade commercially available.

### 3.2. Preparation of Lipophilic Fraction from Liriope platyphylla Seeds

The *L. platyphylla* fruits were obtained from a Duk-in farm in Miryang (Gyeongnam, Republic of Korea). After removing pericarp, the seeds of the fruits were dried at 60 °C. Dried powders of seed were extracted with 95% ethanol twice (24 h/time). After filtration, the filtrates were collected and condensed using a rotary evaporator (EYELA, Japan) to harvest a seed ethanol extract (SEE). The SEE was further fractionated with n-hexane (Hex), and the Hex fraction was concentrated using a rotary evaporator to obtain the lipophilic fraction of *L. platyphylla* seeds (LLPS). The LLPS was stored in amber vials at −80 °C until used.

### 3.3. Chemical Composition Analysis of LLPS

The chemical composition of LLPS was analyzed on an Agilent 7890A gas chromatography instrument (Agilent Technologies, Santa Cruz, CA, USA) coupled with an Agilent 5975C mass selective detector (Agilent Technologies). The lipophilic compounds were separated on a J&W BD-5ms column (60 m × 0.25 mm × 0.25 µm). The column temperature was programmed as follows: initially 50 °C for 5 min, increased to 310 °C at a rate of 10 °C/min, and finally 310 °C for 10 min. The carrier gas was helium with a flow rate of 1 mL/min and pressure of 19.909 psi. The mass spectra were determined between 30 and 500 *m*/*z*, with ionization voltage of 70 eV and a full scan at the rate of 0.132 s/scan. Compounds in LLPS were identified by comparison to mass spectra in the NIST and Wiley mass spectral libraries.

### 3.4. Chemical-Based Antioxidant Activity Assays

#### 3.4.1. Radical Scavenging Assays

The DPPH radical scavenging activity was assayed following the method outlined in a previous study [68]. Various concentrations of the samples were incubated with 0.2 mM of DPPH solution at room temperature in the dark. After 30 min, the absorbance was read at 517 nm using a microplate reader (BioTek, Winnoski, VT, USA).

The ABTS^+^ radical scavenging capacity was assayed following the method outlined in a previous study [68]. The samples at various concentrations were incubated with ABTS^+^ solution at room temperature for 5 min. Absorbance was recorded at 734 nm using a microplate reader (BioTek).

The NO radical scavenging assay was measured following a previous study with minor modifications [58]. The samples at various concentrations were incubated with 10 mM of SNP for 180 min, and the NO level was measured using the Griess reagent. Absorbance was read at 546 nm using a microplate reader (BioTek).

The superoxide radical scavenging activity was carried out following a previous method with minor modifications [62]. The samples at different concentrations were added to a reaction mixture containing NBT and xanthine oxidase. Superoxide radical scavenging capacity was measured spectrophotometrically at 560 nm using a microplate reader (BioTek).

The hydroxyl radical scavenging activity was quantified following a previous method with slight modifications [62]. A reaction mixture containing 40 mM of salicylic acid, 1.5 mM of FeCl_2_, and 60 mM of H_2_O_2_ was incubated with a sample at 37 °C for 1 h. Absorbance was recorded at 510 nm using a microplate reader (BioTek).

The H_2_O_2_ scavenging activity was analyzed using a chemiluminescence method as described in a previous study [69]. Chemiluminescence was measured with a microplate luminometer (BioTek).

Radical scavenging activity was calculated using the following equation.

Scavenging activity (%) = [1 − (Absorbance of sample/Absorbance of control)] × 100

#### 3.4.2. Lipid Peroxidation

Lipid peroxidation was determined using the TBA method [58]. The samples at various concentrations were incubated with lipid emulsion at 40 °C for 48 h to facilitate lipid peroxidation. Then, reaction mixtures were mixed with 1% TBA and 10% trichloroacetic acid and heated in a 95 °C water bath for 1 h. After cooling, absorbance was read at 532 nm using a microplate reader (BioTek). Trolox was utilized as a positive control. The percent inhibition of lipid peroxidation was calculated using the following equation: % inhibition of lipid peroxidation = [1 − (Absorbance of sample/Absorbance of control)] × 100.

#### 3.4.3. Ferric Reducing Antioxidant Power (FRAP) Assay

The FRAP was estimated using a slightly modified procedure as outlined in a previous study [70]. The samples were mixed with the FRAP reagent containing acetate buffer (300 mM, pH 3.6), ferric chloride solution (20 mM), and TPTZ (10 mM). The mixtures were left to react in the dark for 30 min, and absorbance was read at 590 nm. The FRAP value was calculated based on a Trolox standard curve and displayed as the equivalent of Trolox (mg TE/g dried extract).

#### 3.4.4. Potassium Ferricyanide Reducing Antioxidant Power (PFRAP) Assay

The PFRAP was estimated using a previously outlined method with minor modifications [71]. The samples were mixed with 1% potassium ferricyanide. After incubation at 50 °C for 30 min, 10% trichloroacetic acid and 0.1% ferric chloride were added, and absorbance was measured at 700 nm. The PFRAP value was determined using a Trolox standard curve and displayed as the equivalent of Trolox (mg TE/g dried extract).

#### 3.4.5. Total Antioxidant Capacity (TAC) Assay

The TAC was determined following the phosphomolybdenum method [58]. The samples were mixed with 0.6 M of sulfuric acid, 28 mM of sodium phosphate, and 4 mM of ammonium molybdate, and the mixtures were then heated at 95 °C for 90 min. After cooling, absorbance was read at 695 nm using a microplate reader (BioTek). The TAC value was calculated using a Trolox standard curve and displayed as the equivalent of Trolox (mg TE/g dried extract).

### 3.5. Cell Culture

Murine RAW 264.7 monocyte–macrophage cells, purchased from the American Type Culture Collection (Manassas, VA, USA), were nourished in Dulbecco’s modified Eagle’s medium added with 10% fetal bovine serum, 100 U/mL penicillin, and 100 µg/mL streptomycin at 37 °C in a humidified incubator with 5% CO_2_.

### 3.6. Intracellular ROS Formation

RAW 264.7 cells were seeded in a 96-well plate and incubated for 24 h. After pretreatment with LLPS (10 or 100 µg/mL) for 1 h, the cells were challenged with LPS (1 µg/mL) for an additional 6 h. Afterward, the cells were incubated with 20 µM of DCFH-DA in the dark at 37 °C for 1 h. After washing off the probe, the production of intracellular ROS was measured at the wavelengths of 282/20 nm excitation and 528/20 nm emission with a microplate reader (BioTek).

### 3.7. NO Production

The NO level in culture media was determined by the Griess reagent. After treatment, the culture medium was incubated with an equal amount of the Griess reagent for 10 min, and absorbance was read at 546 nm using a microplate reader (BioTek). The NO concentration was quantified based on a standard curve of NaNO_2_.

### 3.8. Western Blotting

Treated cells were homogenized in RIPA buffer (Thermo Scientific, Madison, WI, USA) and incubated on ice for 1 h. After centrifugation, total protein content was quantified using a BCA protein assay kit (Thermo Scientific). Equal amounts of protein were separated on SDS–PAGE gels and transferred onto PVDF. The membranes were blocked with 5% milk in TBST (0.1% Tween 20 in Tris-buffered saline), followed by incubation with specific primary antibodies at 4 °C for 24 h. After washing, the membranes were hybridized with appropriate secondary antibodies at 4 °C for 3 h. Eventually, blots were visualized using a wsE-7120 Ez WestLumi plus reagent (ATTO, Tokyo, Japan).

### 3.9. Network Pharmacology Analysis

The canonical SMILES structures of lipophilic compounds in LLPS were collected from the PubChem database (https://pubchem.ncbi.nlm.nih.gov/, accessed on 1 June 2023). The potential targets of bioactive compounds were predicted using SwissTargetPrediction (https://swisstargetprediction.ch/, accessed on 1 June 2023) [72] and SuperPred (https://prediction.charite.de/, accessed on 1 June 2023) [73] webtools. The collected targets were subjected to the UniProt database (https://www.uniprot.org/, accessed on 1 June 2023) for standardizing gene names. The GeneCards database (https://www.genecards.org/, accessed on 1 June 2023), Online Mendelian Inheritance in Man (OMIM) database (https://www.omim.org/, accessed on 1 June 2023), and Comparative Toxicogenomics database (http://ctdbase.org/, accessed on 1 June 2023) were utilized to screen out target genes related to inflammation, anti-inflammation, oxidation, and antioxidant.

A Venn diagram was generated using bioinformatic webtools (https://bioinformatics.psb.ugent.be/webtools/Venn/, accessed on 2 June 2023) to identify overlapping genes between LLPS compounds-, antioxidant-, and inflammation-target genes. These genes were then interconnected with the compounds to generate the compound–target network, which was visualized and analyzed using Cytoscape 3.9.1 software. Bioactive compounds and genes targets were represented by the nodes, while their interaction was explained by the edges. The 20 most important target nodes were then selected to analyze the protein–protein interaction network through the STRING database (https://string-db.org/, accessed on 2 June 2023). The top 10 hub genes were then determined from the PPI network using the maximal clique centrality (MCC) method of CytoHubba in Cytoscape. The degree value was reflected by the color and the size of the node. The functional properties of LLPS were analyzed through the enrichment of the common genes using Database for Annotation, Visualization, and Integrated Discovery (DAVID, https://david.ncifcrf.gov/, accessed on 2 June 2023). The most important enriched Gene Ontology (GO) and Kyoto Encyclopedia of Genes and Genomes (KEGG) results were visualized through the SRPlot platform (http://www.bioinformatics.com.cn/, accessed on 2 June 2023).

### 3.10. Molecular Docking Analysis

The 3D conformations of potential bioactive compounds were acquired from the PubChem database and charged with Gasteiger partial charges using OpenBabel 2.4.1. The structures of the target proteins were retrieved from the Protein Data Bank (https://www.rcsb.org/, accessed on 5 June 2023). After removing water molecules, adding polar hydrogen, and calculating Kollman charges, simulation docking of bioactive compounds and target proteins was performed using AutoDock Vina 1.5.6 software. The best docking poses were visualized by Discovery Studio 2021 software.

### 3.11. Statistical Analysis

Data are displayed as the mean ± standard deviation (SD). Mean comparisons were executed using the one-way analysis of variance followed by Tukey’s post hoc test. The difference with a *p*-value less than 0.05 was considered statistically significant.

## 4. Conclusions

This study demonstrated that LLPS harbored a powerful antioxidant activity, as evident from the in vitro chemical- and cell-based assays. Also, LLPS exerted anti-inflammatory ability by reducing the levels of pro-inflammatory cytokines and mediators in LPS-stimulated RAW 264.7 cells. Network pharmacology and molecular docking analyses shed light on the underlying mechanisms of LLPS, revealing that bioactive compounds, primarily phytosterols and fatty acid esters, affected core targets, including NFKB1, PTGS1, PTGS2, TLR4, PRKCA, PRKCD, KEAP1, NFE2L2, and NR1l2. Most enriched pathways, such as the TLR, PI3k-Akt, MAPK, NF-κB, and HIF-1 signaling pathways, were associated with inflammation and oxidative stress, underscoring their pivotal roles in antioxidant and anti-inflammatory effects of LLPS. However, this study has limitations, notably the absence of molecular dynamics simulations and binding energy calculations, which could have provided a more detailed understanding of ligand–protein interactions and binding energetics. Further research should address these shortcomings and comprehensively investigate LLPS’s antioxidant and anti-inflammatory properties and its underlying mechanisms in specific models. In conclusion, these data suggest that LLPS may be a promising agent for developing nutraceuticals and functional foods with antioxidant and anti-inflammatory capacities. 

## Figures and Tables

**Table 1:** Chemical compositions of LLPS using GC–MS analysis.

Group	Retention Time	Compound Names	Peak Area (%)
Fatty acids and esters (n = 20)	35.656	Diisobutyl adipate	0.64
	42.14	Ethyl palmitate	3.12
	44.085	11,14-Octadecadienoic acid, methyl ester	0.23
	44.198	8-Octadecenoic acid, methyl ester	0.34
	44.812	Linoleic acid	1.56
	44.919	Oleic acid	6.65
	45.298	Ethyl linoleate	5.45
	45.402	9-Octadecenoic acid ethyl ester	4.24
	45.646	Hexadecanamide	0.33
	45.831	Ethyl stearate	0.55
	47.629	Glycidyl palmitate	2.12
	48.708	Ethyl stearate, mono 9-epoxy	3.38
	48.789	9-Octadecenamide	1.48
	49.226	Butanoic acid, [2-(1-hexenyl)cyclopropyl]methyl ester, [1R-[1.alpha.,2.alpha.(E)]]-	2.11
	50.509	Butyl linoleate	2.93
	50.575	Glycidyl oleate	3.62
	51.143	2-Palmitoylglycerol	3.61
	55.236	Ethyl tetracosanoate	0.29
	53.917	Glyceryl monooleate	20.05
	54.24	Glyceryl monostearate	1.03
Hydrocarbons (n = 7)	10.229	Hexanal	0.94
	26.055	(E,Z)-2,4-Decadienal	0.11
	26.735	2,4-Decadienal	0.1
	30.921	6,7-Dimethyltetralin-1,5,8-trione	0.2
	48.002	Cyclopropaneoctanal, 2-octyl-	0.58
	48.458	7-Pentadecyne	0.74
	49.786	1,3,12-Nonadecatriene	0.35
Phytosterols (n = 4)	64.459	Stigmasterol	1.64
	66.102	Clionasterol	6.95
	66.503	Fucosterol	1.97
	68.598	Cycloartenol	1.31
Terpenes (n = 4)	32.514	Alpha-selinene	0.74
	40.677	3-Buten-2-one, 3-methyl-4-(2,6,6-trimethyl-1-cyclohexen-1-yl)-	0.31
	55.608	Squalene	1.57
	57.458	2,3-Oxidosqualene	0.47
Tocols (n = 1)	63.822	Tocotrienol, alpha	1.87
Others (n = 7)	16.581	2-Pentylfuran	0.12
	19.678	2,3,5,6-Tetramethylpyrazine	0.24
	34.231	2-Methoxy-3-(tert-butyl)-5-methylphenol	5.14
	36.095	2-Oxatricyclo[4.3.1.0(3,8)]decane	0.22
	46.232	3,6-Diazahomoadamantan-9-ol	0.55
	51.476	Bis(2-ethylhexyl) phthalate	0.24
	53.184	3-n-Butylthiophene-1,1-dioxide	0.67
Fatty acids and esters			63.73
Hydrocarbons			3.02
Phytosterols			11.87
Terpenes			3.09
Tocols			1.87
Others			7.18
**Total**			**90.76**

**Table 2:** Reducing antioxidant powers and total antioxidant capacity of LLPS.

	FRAP (mg TE/g)	PFRAP (mg TE/g)	TAC (mg TE/g)
LLPS	40.71 ± 1.28	25.55 ± 0.94	104.55 ± 4.69

FRAP: Ferric reducing antioxidant power; PFRAP: Potassium ferricyanide reducing antioxidant power; TAC: Total antioxidant capacity; LLPS: Lipophilic fraction from *L. platyphylla* seeds.

## Data Availability

The data, analytic methods, and study materials that support the findings of this study are available from the corresponding author upon reasonable request.

## Supplementary Materials

The following supporting information can be downloaded at: https://www.mdpi.com/article/10.3390/ijms241914958/s1.
