# Supplementary material for: Anti-Inflammatory and Antioxidant Activities of Lipophilic Fraction from Liriope platyphylla Seeds Using Network Pharmacology, Molecular Docking, and In Vitro Experiments
Source: Int J Mol Sci. 2023 Oct 6;24(19):14958. doi: 10.3390/ijms241914958 (PMC10573744; doi:10.3390/ijms241914958)
Supplement: Supplementary file 1 [file ijms-24-14958-s001.zip › ijms-2611677-supplementary.pdf]

International Journal of
Molecular Sciences

Supplementary Materials

Supplementary Figure S1. Compounds-targets network: green triangle nodes are compounds and red shades octagonal nodes are targets.

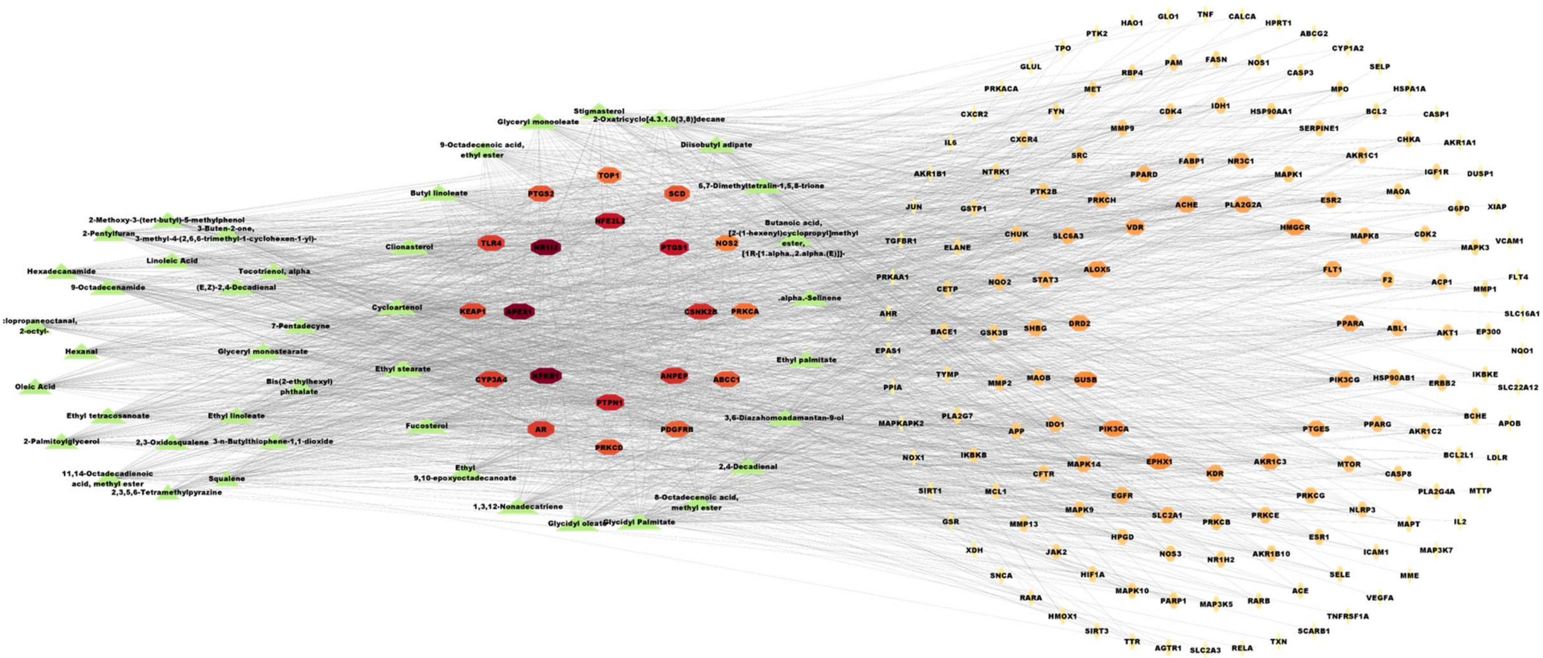

International Journal of *Molecular Sciences*

MDPI

Supplementary Table S1. Molecular docking results of the top 10 Hub genes and the 20 active compounds in LLPS.

Proteins	Compounds	Binding affinity (kcal/mol)	Interaction bonds	
			H-bonds/Covalent bonds/ Alkyl bonds/ Pi-sigma bonds/ Pi-Pi stacked/ Unfavorable acceptor-acceptor/ Unfavorable donor-donor	Van der Waals
NOS2 (4NOS)	Stigmasterol	-10.9	Trp194, Ala197, Arg199, Cys200, Met355, Phe369, Trp372, Tyr489	Pro198, Ile201, Gly202, Gln205, Ser242, Val352, Gly371, Met374, Glu377, Phe488
	Cycloartenol	-10.4	Trp194, Leu209, Ile244, Gln263, Phe369, Tyr489	Cys200, Ser242, Pro350, Ala351, Val352, Asn370, Gly371, Tyr373, Glu377, Aso382, Arg388
	Clionasterol	-10.1	Ala197, Arg199, Cys200, Met355, Phe369, Trp372, Tyr489	Trp194, Pro198, Ile201, Gly202, Gln205, Ser242, Val352, Gly371, Met374, Glu377, Phe488, Tyr490
	Fucosterol	-9.1	Ala197, Arg199, Val352, Met355, Arg381, Trp463, Pro467	Pro198, Cys200, Ile201, Phe369, Met374, Thr376, Glu377, Tyr486, Tyr491
	6,7-Dimethyltetralin-1,5,8-trione	-7.7	Trp194, Cys200, Phe369	Ala197, Leu209, Ser242, Met355, Asn370, Gly371, Tyr489
	alpha-Selinene	-7.3	Trp194, Cys200, Phe369	Gly202, Leu209, Ser242, Val352, Asn370, Gly371, Trp372, Tyr489
	Glyceryl monooleate	-7.0	Leu125, Trp194, Ala197, Pro198, Arg199, Cys200, Pro350, Val352, Met355, Phe369, Phe488, Tyr489, Tyr491	Gly202, Leu209, Ser242, Ala351, Asn370, Gly371, Trp372, Tyr490
	Butyl linoleate	-7.0	Leu125, Trp194, Ala197, Pro198, Arg199, Cys200, Leu209, Pro350, Val352, Met355, Phe369, Phe488, Tyr489, Tyr491	Ser242, Ala351, Asn370, Gly371, Trp372, Tyr490
	Butanoic acid, [2-(1-hexenyl)cyclopropyl]methyl ester, [1R-[1.alpha.,2.alpha.(E)]]-	-7.1	Leu125, Trp194, Ala197, Cys200, Met355, Phe369, Phe488, Tyr489, Tyr491	Pro198, Arg199, Leu209, Ser242, Asn370, Gly371, Tyr490
	Glycidyl oleate	-7.1	Leu125, Trp194, Ala197, Arg199, Cys200, Leu209, Met355, Phe369, Gly371 Phe488, Tyr489, Tyr491	Ser242, Pro350, Ala351, Val352, Asn370, Glu377, Tyr490

	1,3,12-Nonadecatriene	-6.5	Trp194, Leu209, Pro350, Phe369, Trp372, Tyr489	Ile201, Gly202, Gln205, Ser242, Ala351, Val352, Asn370, Gly371, Glu377
	Ethyl stearate	-6.2	Leu125, Trp194, Ala197, Arg199, Cys200, Phe369, Tyr489, Tyr491	Pro198, Leu209, Ser242, Val352, Met355, Asn370, Gly371, Phe488
	Glycidyl palmitate	-6.3	Trp194, Cys200, Leu209, Pro350, Val352, Phe369, Tyr373, Tyr489	Ser242, Ile244, Gln263, Arg266, Tyr347, Ala351, Asn370, Gly371, Trp372 Glu377, Asp382, Arg388
	Ethyl 9,10-epoxyoctadecanoate	-6.4	Leu125, Trp194, Ala197, Pro198, Arg199, Cys200, Leu209, Pro350, Phe369, Phe488, Tyr489, Tyr491	Ser242, Val352, Met355, Asn370, Gly371, Trp372, Glu377
	9-Octadecenoic acid, ethyl ester	-6.5	Leu125, Trp194, Ala197, Arg199, Cys200, Leu209, Phe369, Phe488, Tyr489, Tyr491	Pro198, Ser242, Val352, Met355, Asn370, Gly371, Glu377
	Diisobutyl adipate	-6.3	Leu125, Trp194, Pro198, Arg199, Phe369, Phe488, Tyr489, Tyr491	Ala197, Cys200, Leu209, Val352, Met355, Asn370, Gly371
	Ethyl palmitate	-6.1	Leu125, Trp194, Ala197, Pro198, Arg199, Cys200, Gly202, Met355, Phe369, Trp372, Phe488, Tyr491	Gln205, Gly371, Tyr489, Tyr490
	2,4-Decadienal	-5.9	Trp194, Ala197, Cys200, Leu209, Phe369, Tyr489	Arg199, Ser242, Asn370, Gly371
	3,6-Diazahomoadamantan-9-ol	-5.6	Gly253, Lys254, Asp256, Arg258, Gln310	Pro129, Gly313, Ala495, Thr498, His499, Val500
	2-Oxatricyclo[4.3.1.0(3,8)]decane	-5.2	Gln310, Gly313, His499	Pro129, Gly253, Lys254, Asp256, Arg258, Ala495, Thr498, Val500
KEAP1 (6TYP)	Stigmasterol	-9.9	Ala366, Arg415, Val465, Ala556, Val512	Tyr334, Ser363, Gly364, Leu365, Gly367, Arg380, Asn414, Gly462, Val463, Gly464, Ala466, Gly509, Ala510, Gly511, Cys513, Phe577, Ile559, Gly603, Val604, Gly605, Val606
	Cycloartenol	-10.1	ALa366, Gly367, Arg415, Tyr525, Ala556	Gly364, Leu365, Ile416, Val418, Gly462, Gly464, Val465, Ser508, Gly509, Ala510, Gly511, Val512, Ser555, Leu557, Gly558, Ile559, Gly603, Val604, Gly605, Val606
	Clionasterol	-9.2	Ala466, Cys513, Ala556	Gly364, Leu365. Ala366, Gly367, Arg415, Val418, Gly419, Gly462, Val463, Gly464, Val465, Val467, Gly509, Ala510, Gly511, Val512, Val514, Ile557, Gly558, Ile559, Thr560, Gky603, Val604, Val606, Ala607

Fucosterol	-8.3	Val465, Val467	Gly367, Cys368, Val418, Gly419, Val420, Ala466, Val512, Cys513, Val514, Ile559, Val561, Thr560, Ala607
6,7-Dimethyltetralin-1,5,8-trione	-7.7	Tyr334, Ser363, Arg380, Ser602	Gly364, Asn382, Arg415, Ala556, Tyr572, Phe577, Gly603
alpha-Selinene	-6.8	Ala366	Leu365, Gly376, Ile416, Gly417, Val418, Gly462, Val463, Gly464, Val465, Gly509, Ala510, Gly511, Leu557, Gly558, Ile559, Val604, Gly605, Val606
Glyceryl monooleate	-6.3	Tyr334, Ile416, Val463, Tyr525, Ala556, Tyr572, Phe577	Ser363, Gly364, Leu365, Ala366, Arg380, ARg415, Gly417, Gly462, Gly464, Gly509, Gln530, Ser555, Ser602, Gly603
Butyl linoleate	-6.0	Tyr334, Arg380, Arg415, Tyr525, Ala556, Tyr572	Ser363, Gly364, Asn414, Ser508, Gly509, Gln530, Ser555, Phe577, Ser602, Gly603
Butanoic acid, [2-(1-hexenyl)cyclopropyl]methyl ester, [1R-[1.alpha.,2.alpha.(E)]]-	-5.4	Arg415, Ser508, Ala556, Tyr572, Phe577	Tyr334, Gly462, Arg483, Gly509, Tyr525, Ser555, Ser602
Glycidyl oleate	-5.9	Tyr334, Arg415, Tyr525, Ala556, Tyr572, Phe577	Ser363, Gly364, Arg380, Gly462, Gly509, Gln530, Ser555, Ser602, Gly603
1,3,12-Nonadecatriene	-5.3	Tyr334, Arg415, Tyr525, Ala556, Tyr572, Phe577	Ser363, Gly364, Arg380, Gly509, Gln530, Ser555, Ser602, Gy603
Ethyl stearate	-5.7	Tyr334, Ser363, Arg380, Tyr525, Ala556, Tyr572	Gly364, Arg415, Gly509, Gln530, Ser555, Phe577, Ser602, Gly603
Glycidyl palmitate	-5.6	Tyr334, Asn414, Tyr525, Ala556, Tyr572, Phe577	Ser363, Gly364, Arg380, Arg415, Gln530, Ser555, Ser602
Ethyl 9,10-epoxyoctadecanoate	-5.8	Tyr525, Ser555, Ala556, Tyr572	Tyr334, Ser363, Gly364, Arg380, Asn414, Ile461, Gly462, Arg483, Ser508, Gly509, Phe577, Ser602, Gly603
9-Octadecenoic acid, ethyl ester	-5.6	Tyr334, Arg380, Arg415, Tyr525, Ala556, Tyr572, Phe577	Ser363, Gly364, Asn414, Gln530, Ser555, Ser602, Gly603
Diisobutyl adipate	-5.6	Gly367, Ala556, Gly605, Val606	Gly364, Leu365, Arg415, Ile416, Gly417, Val418, Gly462, Val463, Gly464, Val465, Gly509, Ala510, Val512, Leu556, Leu557 Ile559, Gly603, Val604

	Ethyl palmitate	-5.8	Tyr334, Arg380, Arg415, Tyr525, Tyr572, Phe577	Ser363, Gly364, Asn414, Arg483, Ser508, Gly509, Gln530, Ser555, Ala556, Ser602
	2,4-Decadienal	-5.0	Tyr334, Asn414, Arg415, Ala556, Tyr572, Phe577	Ser363, Gly364, Arg380, Ser555, Ser602
	3,6-Diazahomoadamantan-9-ol	-6.2	Leu365, Gly367, Leu557, Ile559, Val606	Ala366, Cys368, Gly511, Val512, Gly558, Val604, Gly605, Ala607
	2-Oxatricyclo[4.3.1.0(3,8)]decane	-5.2	Tyr334, Arg415, Ala556	Ser363, Gly364, Arg380, Ser602, Gly603
PTGS2 (5IKQ)	Stigmasterol	-8.9	Leu294, Leu391, Tyr404, Ile408, Ala443, Val444	Ala202, Gln203, His207, Tyr385, His386, Trp387, His388, Leu390, Val447
	Cycloartenol	-8.7	Tyr385, Trp387, Tyr404, Ile408	Gln203, His207, Leu294, Val295, His386, His388, Leu390, Leu391, Gln405, Val444, Val447
	Clionasterol	-8.9	Leu294, Leu391, Ile408	Ala202, Gln203, Thr206, His207, Tyr385, His386, Trp387, His388, Leu390, Tyr404, Ala443, Val444, Val447
	Fucosterol	-8.7	His207, Lys211, Gln289, Val291, His388, Leu391	Ala202, Gln203, Thr206, Phe210, Thr212, His214, Tyr385, His386, Trp387, Leu390, Val447
	6,7-Dimethyltetralin-1,5,8-trione	-7.8	His207, His388	Ala199, Ala202, Gln203, Thr206, Phe210, Asn382, Tyr385, His386, Trp387, Leu390, Leu391
	alpha-Selinene	-8.0	Val349, Leu352, Val523, Ala527	Tyr348, Ser353, Tyr385, Trp387, Phe518, Met522, Gly526, Ser530
	Glyceryl monooleate	-6.8	Val349, Leu352, Ser353, Tyr385, Trp387, Phe518, Met522, Val523, Ala527, Leu531	His90, Val116, Arg120, Gln192, Tyr348, Gly354, Tyr355, Leu359, Phe381, Leu384, Arg513, Ala516, Ile517, Gly526, Ser530,
	Butyl linoleate	-6.7	His90, Val523, Val349, Tyr355, Tyr385, Trp387, Ala516, Ile517, Phe518, Met522, Val523, Ala527, Leu531	Val116, Arg120, Gln192, Tyr348, Leu352, Ser353, Leu359, Leu384, Arg513, Gly526, Ser530
	Butanoic acid, [2-(1-hexenyl)cyclopropyl]methyl ester, [1R-[1.alpha.,2.alpha.(E)]]-	-6.8	Val116, Arg120, Val349, Leu352, Tyr355, Leu359, Tyr385, Trp387, Phe518, Val523, Leu531	Met113, Tyr348, Ser353, Phe381, Leu384, MEt522, Gly526, Ala527, Ser530,
	Glycidyl oleate	-6.8	Arg120, Val349, Leu352, Tyr355, Leu384, Tyr385, Trp387, Phe518, Val523, Ala527	Val89, Leu93, Tyr115, Val116, Ser119, Tyr348, Ser353, Phe381, Met522, Gly526, Ser530, Leu531
	1,3,12-Nonadecatriene	-6.9	Val116, Ile345, Val349, Leu352, Tyr355, Leu359, Tyr385, Trp387, Phe518, Val523, Ala527, Leu531	Met113, Arg120, Trp348, Ser353, Phe381, Leu384, Met522, Gly526, Ser530

	Ethyl stearate	-5.9	Ala202, Val295, His388, Leu390, Leu391, Phe395, Tyr404, Phe407, Ile408, Val444, Val447	Ala199, Phe200, Gln203, Thr206, His207, Phe210, Leu294, Tyr385, His386, Trp387
	Glycidyl palmitate	-6.1	Val349, Leu352, Tyr355, Tyr385, Trp387, Ala527, Leu531	Val116, Arg120, Tyr348, Ser353, Leu359, Phe381, Leu384, Phe518, Met522, Val523 Gly526, Ser530
	Ethyl 9,10-epoxyoctadecanoate	-6.8	Val116, Ile345, Val349, Leu352, Tyr355, Leu359, Leu384, Tyr385, Trp387, Phe518, Met522, Val523, Ala527, Ser530, Leu531	Met113, Arg120, Tyr348, Ser353, Phe381, Glt526
	9-Octadecenoic acid, ethyl ester	-6.7	Arg120, Ile345, Val349, Leu352, Tyr355, Leu359, Leu384, Tyr385, Trp387, Phe518, Met522, Val523, Ala527, Leu531	Met113, Val116, Tyr348, Ser353, Phe381, Gly526, Ser530
	Diisobutyl adipate	-6.6	Val116, Tyr348, Val349, Leu352, Tyr355,, Leu359, Leu531	Met113, Arg120, Ser353, Tyr385, Trp387, Phe518, Met522, Val523, Gly526, Ala527, Ser530
	Ethyl palmitate	-6.7	Leu352, Tyr355, Tyr385, Trp387, Phe518, Val523, Ala527, Ser530	Val116, Arg120, Tyr348, Val349, Ser353, Leu359, Phe381, Leu384, Met522, Gly526
	2,4-Decadienal	-5.3	Val349, Leu352, Tyr355, Phe518, Met522, Val523, Ala527	Arg120, Tyr348, Ser353, Phe381, Tyr385, Trp387, Gly526, Ser530
	3,6-Diazahomoadamantan-9-ol	-5.5	Pro84, Ser119, Arg120, Glu524	Lys83, Pro86, Val89, Tyr115, His122
	2-Oxatricyclo[4.3.1.0(3,8)]decane	-5.6	Val349, Leu352, Phe518, Val523, Ala527	Ser353
	Stigmasterol	-8.4	Pro128, Arg374, Arg376, Pro538	Ser126, Pro127, Phe142, Val145, Gly225, His226, Asp229, Arg333, Leu334, Ile337, Asn375, Asn537
	Cycloartenol	-8.2	Leu93, Leu112,	Arg83, Pro84, Pro86, Thr89, Leu92, Trp100, Leu115, Val116, Val119, Arg120, Leu357, Gly471, Glu524
PTGS1 (6Y3C)	Clionasterol	-7.8	Phe356	Gln192, Glu347, Gln350, Gln351, Gly354, Tyr355, Leu564, Val578, Ser579, Phe580, Arg581
	Fucosterol	-7.6	Arg79, Arg83	His43, Gln44, Thr62, Gly63, Tyr64, Thr76, Asn80, Val119, Asn122, Leu123, Gly471
	6,7-Dimethyltetralin-1,5,8-trione	-8.1	Ala202, Gln203, His207, His388	Ala199, Thr206, Phe210, Tyr385, Trp387, Leu390, Met391
	alpha-Selinene	-6.6	His207, His386	Ala202, Gln203, Thr206, Phe210, Asn382, Tyr385, His388, Val447

Glyceryl monooleate	-6.0	Cys36, Tyr39, Cys47, Arg49, Pro153, Pro156	Asn34, Cys41, Gln42, His43, Gln44, Gly45, Val48, Asp135, Tyr136, Leu152, Ser154, Gln461, Glu465, Lys468, Arg469
Butyl linoleate	-5.7	Cys36, Tyr39, Gln44, Gly45, Ile46, Cys47, Pro153	Pro40, Cys41, Gln42, His43, Val48, Arg49, Tyr130, Asp135, Tyr136, Ile137, Leu152, Gln461, Arg469
Butanoic acid, [2-(1-hexenyl)cyclopropyl]methyl ester, [1R-[1.alpha.,2.alpha.(E)]]-	-6.0	Phe205, Phe209, Val344, Tyr348, Val349, Leu352, Tyr385, Trp387, Leu534	Ser353, Phe381, Leu384, Phe518, Met522, Ile523, Gly526, Ala527, Ser530, Gly533,
Glycidyl oleate	-6.2	Cys36, Tyr39, Ile46, Cys47, Tyr130, Pro153, Pro156, Glu465, Lys468	Pro40, Cys41, Gln42, His43, Gln44, Gly45, Asp135, Leu152, Ser154, Gln461, Arg469
1,3,12-Nonadecatriene	-6.5	Ala199, Ala202, Phe210, Leu295, Tyr385, His386, Trp387, His388, Leu390, Met391, Phe395, Tyr404, Phe407, Leu408, Ile444	Gln203, Thr206, Asn382
Ethyl stearate	-7.2	Arg120, Phe205, Phe209, Val228, Val344, Tyr348, Val349, Leu352, Ser353, Tyr355, Leu359, Ile377, Phe381, Trp387, Phe518, Ile523, Ala527, Leu531, Leu534	Val116, Gly227, Asn375, Tyr385, Met522, Gly526, Ser530, Gly533
Glycidyl palmitate	-5.9	Phe205, Gly227, Tyr348, Val349, Leu352, Ile377, Phe381, Tyr385, Trp387, Phe518, Ile523, Ala527, Leu534	Arg120, Phe209, Val228, Val344, Ser353, Asn375, Arg376, Ala378, Leu384, Gly526, Phe529, Ser530, Gly533
Ethyl 9,10-epoxyoctadecanoate	-6.4	Phe205, Val344, Tyr348, Val349, Leu352, Tyr355, Leu359, Ile377, Phe381, Tyr385, Ile523, Ala527, Phe529, Ser530, Leu534	Arg120, Phe209, Gly227, Val228, Ser353, Asn375, Trp387, Phe518, Met522, Gly526, Gly533
9-Octadecenoic acid, ethyl ester	-6.2	Val116, Phe205, Phe209, Val344, Tyr348, Val349, Leu352, Tyr355, Leu359, Ile377, Tyr385, Trp387, Ile523, Ala527, Ser530, Leu531, Gly533, Leu534,	Arg120, Gly227, Val228, Ser353, Asn375, Phe381, Leu384, Phe518, Met522, Gly526, Phe529
Diisobutyl adipate	-6.1	Cys36, Cys41, Pro153, Pro156, Gln461, Lys468	Tyr39, Gln42, His43, Gln44, Gly45, Cys47, Asp135, Leu152, Ser154, Val155, Glu465, Arg469
Ethyl palmitate	-5.7	Cys36, Tyr39, Gln44, Gly45, Ile46, Cys47, Tyr130, Pro153, Pro156, Lys468, Arg469	Cys41, Gln42, His43, Asp135, Leu152, Ser154, Gln461, Glu465
2,4-Decadienal	-5.3	Arg120, Val349, Leu352, Tyr355, Trp387, Phe518, Ala527	Tyr348m Ser353, Met522, Ile523, Gly526, Ser530,

	3,6-Diazahomoadamantan-9-ol	-5.5	Ile523	Val349, Leu352, Ser353, Leu384, Trp387, Phe518, Met522, Gly526, Ala527, Ser530
	2-Oxatricyclo[4.3.1.0(3,8)]decane	-6.3	Val349, Leu352, Phe518, Ile523, Ala527	Ser353, Trp387, Ser530, Gly526
NR1I2 (6P2B)	Stigmasterol	-7.8	Lys170, Leu174, Leu213, Leu215, Trp223	Phe172, Pro175, Val177, Gly217, Glu218, Asn224, Tyr225, Arg303
	Cycloartenol	-8.1	Phe172, Leu174, Leu215, Trp223	Pro175, Gly176, Val177, Asn224, Tyr225, Lys226
	Clionasterol	-7.1	Trp223, Tyr225	Leu174, Pro175, Gly176, Val177, Leu215, Asn224, Lys226, Pro228
	Fucosterol	-6.8	Lys277, Pro354, Ala405	Ser274, Gly278, Arg353, Asn404, Thr408, Gln409, Leu412, Phe429, Gly430
	6,7-Dimethyltetralin-1,5,8-trione	-7.3	Val211, Trp299	Leu209, Met243, Gln285, Phe288, Tyr306, Met323, Leu324, His327
	alpha-Selinene	-7.9	Phe288, Trp299, Tyr306	Leu209, Val211, Met243, Met246, Gln285, Met323, His327, His407
	Glyceryl monooleate	-6.2	Leu209, Lys210, Val211, Met243, Phe288, Trp299, Tyr306	Leu206, Leu239, Leu240, Met246, Ser247, Gln285, Met323, His327, His407
	Butyl linoleate	-6.7	Val211, Leu240, Met243, Ser247, Phe288, Trp299, Tyr306, His327	Leu206, Leu209, Ala244, Gln285, Leu308, Met323, Leu324, His407, Leu411, Ile414, Phe420, Met425,
	Butanoic acid, [2-(1-hexenyl)cyclopropyl]methyl ester, [1R-[1.alpha.,2.alpha.(E)]]-	-6.2	Met243, Cys284, Phe288, Trp299, Tyr306, His407, Leu411, Met425, Phe429	Met246, Ser247, Phe251, Phe281, Gln285, Met323, His327
	Glycidyl oleate	-6.2	Leu209, Val211, Met243, Met246, Phe281, Phe288, Trp299, Tyr306, Met323, His327, His407	Leu206, Leu240, Ser247, Cys284, Gln285, Leu324, Ile414, Phe420
	1,3,12-Nonadecatriene	-5.9	Leu209, Val211, Met243, Phe288, Trp299, Tyr306, Met323, His327, His407	Leu240, Ser247, Gln285, Leu308, Leu324
	Ethyl stearate	-5.6	Leu209, Val211, Leu240, Met243, Ser247, Phe288, Trp299, Tyr306, His407, Phe420	Gln285, Met323, His327, Leu411, Met425
	Glycidyl palmitate	-6.2	Leu209, Val211, Met243, Gln285, Phe288, Trp299, Tyr306, Met323, His407	Ser247, Phe281, Cys284, Leu308, Leu324, His327, Leu411, Phe420, Met425
	Ethyl 9,10-epoxyoctadecanoate	-6.3	Leu209, Val211, Met243, Gln285, Phe288, Trp299, Tyr306, Met323, His407	Leu206, Leu240, Leu324, Arg410, Leu411, Ile414, Phe420
	9-Octadecenoic acid, ethyl ester	-5.6	Leu209, Val211, Met243, Phe288, Trp299, Tyr306, Met323, His327, His407	Leu206, Leu240, Met246, Ser247, Gln285, Leu324

	Diisobutyl adipate	-6.3	Leu206, Leu240, Ser247, Gln285, Phe288, Trp299, His407, Leu411, Ile414	Met243, Ala244, Cys284, His327, Phe420, Met425
	Ethyl palmitate	-5.9	Leu209, Val211, Met243, Ser247, Phe288, Trp299, Tyr306, Leu308, Leu324, His407	Aka244, Phe281, Gln285, Met323, His327, Leu411, Phe420, Met425
	2,4-Decadienal	-5.4	Ser247, Phe288, Trp299, Tyr306, His407	Met243, Met246, Phe281, Gln285
	3,6-Diazahomoadamantan-9-ol	-5.5	His327	Val211, Met243, Gln285, Phe288, Trp299, Tyr306, Met323
	2-Oxatricyclo[4.3.1.0(3,8)]decane	-6.4	Met243, Phe288, Trp299, Tyr306	Val211, Met246, Gln285
PRKCA (6AR4)	Stigmasterol	-7.7	Ile35, Ile37, Phe53, Ala87, Ile90	Leu32, Ile33, Gly34, Thr56, Gln91
	Cycloartenol	-7.3	Leu32, Phe53, Ala87, Ile90	Lys27, Asn31, Ile33, Gly34, Ile35, Ser36, Ile37, Gln91
	Clionasterol	-7.9	Phe53, Ala87	Leu32, Ile33, Gly34, Ile35, Ser36, Ile37, Asp54, Thr56, Val86, Ile90, Gln91
	Fucosterol	-5.8	Leu32, Ile35, Ile37	Gly34, Ser36, Phe53, Lys83, Val84, Ala87, Gln91
	6,7-Dimethyltetralin-1,5,8-trione	-6.1	Leu32, Ile33, Gly34	Ile35, Phe53, Ala87, Ile90, Gln91
	alpha-Selinene	-6.9	Leu32, Ile35, Ile37, Ala87, Ile90	Ile33, Gly34, Ser36, Gln91
	Glyceryl monooleate	-5.4	Asp28, Gln30, Leu32, Ile35, Ile37, Phe53, Thr56 Ala87, Ile90	Ile33, Gly34, Ser36, Asn55, Pro57, Gln91
	Butyl linoleate	-5.7	Leu32, Ile33, Gly34, Ile35, Ile37, Phe53, Lys83, Val84, Ala87, Lys88, Ile90	Lys27, Ser36, Gln91
	Butanoic acid, [2-(1-hexenyl)cyclopropyl]methyl ester, [1R-[1.alpha.,2.alpha.(E)]]-	-5.3	Leu32, Phe53	Ile33, Gly34, Ile35, Ser36, Ile37, Ala87, Ile90, Gln91
	Glycidyl oleate	-5.5	Leu32, Ile35, Ile37, Phe53, Asp54, Ala87, Ile90	Lys27, Asp28, Asn31, Ile33, Gly34, Ser36, Asn55, Thr56, Gln91
	1,3,12-Nonadecatriene	-4.9	Leu32, Ile33, Ile35, Ile37, Phe53, Ala87, Ile90	Gly34, Ser36, Gln91
	Ethyl stearate	-5.6	Leu32, Ile33, Ile35, Ile37 Phe53, Lys83, Val84, Ala87, Ile90	Asp28, Gln30, Gly34, Ser36, Thr56, Gln91
	Glycidyl palmitate	-5.0	Leu32, Ser36, Ile35, Ile37, Phe53, Ala87, Ile90	Ile33, Gly34, Val50, Lys83, Gln91
	Ethyl 9,10-epoxyoctadecanoate	-5.9	Leu32, Ile33, Gly34, Ile35, Ile37, Phe53, Ala87, Ile90	Ser36, Thr56, Gln91
	9-Octadecenoic acid, ethyl ester	-5.3	Gln30, Leu32, Ile33, Ile35, Ile37, Phe53, Ala87, Ile90	Asp28, Gly34, Ser36, Asp54, Asn55, Thr56, Gln91

	Diisobutyl adipate	-5.5	Leu32, Ile33, Gly34, Ile35, Ile37, Phe53, Lys83, Ala87	Ser36, Ile90, Gln91
	Ethyl palmitate	-5.3	Leu32, Ile35, Ile37, Phe53, Ala87, Ile90	Ile33, Gly34, Ser36, Gln91
	2,4-Decadienal	-5.2	Leu32, Ile33, Ile35, Ile37, Ala87, Ile90	Gly34, Ser36, Gln91
	3,6-Diazahomoadamantan-9-ol	-4.0	Asn102	Tyr48, Gly67, Asp68, Glu69, Lys103, Leu104
	2-Oxatricyclo[4.3.1.0(3,8)]decane	-5.2	Leu32, Ile33, Ala87, Ile90	Gly34, Ile35, Ile37, Gln91
PRKCD (1YRK)	Stigmasterol	-7.6	Phe27, Tyr52, Ala77	Gln25, Glu54, Trp55, Met74, Glu78
	Cycloartenol	-7.6	Phe27, Tyr52, Ala77	Gln25, Glu54, Trp55
	Clionasterol	-7.0	Gln25, Phe27, Tyr52, Glu54, Trp55	Glu54, Trp55, Ala77
	Fucosterol	-6.6	Phe27	Gln25, Tyr52, Pro53, Glu54, Trp55, Ala76
	6,7-Dimethyltetralin-1,5,8-trione	-5.9	Phe27	Gln25, Tyr52, Glu54, Trp55
	alpha-Selinene	-5.7	Phe27	Tyr52, Pro53, Glu54, Trp55
	Glyceryl monooleate	-4.8	Phe27, Tyr52, Trp55, Ala77, Glu78	Gln25, Ala29, Lys31, Thr50, Pro53, Glu54, Met74
	Butyl linoleate	-4.2	Phe27, Tyr52, Ala77	Gln25, Pro53, Glu54, Ala76
	Butanoic acid, [2-(1-hexenyl)cyclopropyl]methyl ester, [1R-[1.alpha.,2.alpha.(E)]]-	-4.7	Phe27, Tyr52, Trp55, Ala77	Gln25, Pro53, Glu54
	Glycidyl oleate	-4.4	Phe27, Tyr52, Trp55	Ala23, Gln25, Glu54, Pro53
	1,3,12-Nonadecatriene	-4.3	Phe27 Tyr52, Trp55	Gln25, Pro26, Pro53, Glu54, Ala77
	Ethyl stearate	-4.2	Phe27, Tyr52, Trp55, Ala77	Gln25, Pro26, Cys28, Pro53, Glu54
	Glycidyl palmitate	-4.0	Gln25, Phe27, Tyr52, Glu54, Trp55	Cys28, Pro53
	Ethyl 9,10-epoxyoctadecanoate	-3.9	Phe27, Tyr52, Ala77	Ala23, Asn24, Gln25, Pro53, Glu54, Trp55, Arg75, Ala76
	9-Octadecenoic acid, ethyl ester	-4.0	Phe27, Ala76, Ala77	Als23, Asn24, Gln25, Tyr52, Pro53, Glu54, Trp55, Arg75
	Diisobutyl adipate	-4.1	Phe27, Trp55	Gln25, Pro26, Cys28, Tyr52, Pro53, Glu54, Ala76
	Ethyl palmitate	-3.9	Ala23, Phe27, Tyr52	Gln25, Pro53, Glu54, Trp55
	2,4-Decadienal	-4.2	Phe27, Tyr52, Ala77	Met74, Glu78
	3,6-Diazahomoadamantan-9-ol	-4.0	Ala19, Glu22, Asn24	Gln18, Glu20, Asp21
	2-Oxatricyclo[4.3.1.0(3,8)]decane	-3.9	Phe27	Tyr52, Pro53, Glu54
NFKB1	Stigmasterol	-7.2	Tyr60, Val61, Ala62, Val145	Glu63, Leu143, His144, Thr146, Lys149, Thr153, Arg157

(1SCV)	Cycloartenol	-7.1	Phe56, Arg57, Lys79	Lys52, Gly55, Arg59, His67, Gly68, Gly69, Pro71, Ser74, Ser75, Lys80, Ser81, Ser243
	Clionasterol	-6.7	Pro71, Ser74, Lys79, Lys80	Lys52, Gly55, Phe56, Arg57, Arg59, His67, Gly68, Gly69, Ser75, Ser81
	Fucosterol	-6.9	Val61, Leu143, Val145, Lys149	Pro65, Ser113, Val115, His144, Thr146, Thr153, Arg157
	6,7-Dimethyltetralin-1,5,8-trione	-6.2	Ser113, Val145, Thr153, Arg157	Val61, Leu143, His144, Thr146, Lys149, Ala156
	alpha-Selinene	-5.6	Val61, Val115, Leu143, Val145	Ala111, His112, Ser113, Asp121, His144, Lys149, Thr153, Arg157
	Glyceryl monooleate	-5.5	Arg59, Pro65, Ser113, Gly122, Val115, Leu143, Arg157	Val61, Gly64, His67, Gly68, Gly69, Leu114, Gly116, Glu120, Asp121, Asn139, Gly141, Ile142, Thr153
	Butyl linoleate	-4.9	Arg59, Val61, Pro65, Val115, Leu143	Gly64, Gly68, Gly69, Gky116, Lys117, Asn139, Leu140, Gly141, Ile142
	Butanoic acid, [2-(1-hexenyl)cyclopropyl]methyl ester, [1R-[1.alpha.,2.alpha.(E)]]-	-4.9	Val61, Ala62, Leu143, Val145, Thr153	Tyr60, Ala111, His112, Ser113, His144, Thr146, Lys149, Arg157
	Glycidyl oleate	-4.8	Tyr60, Val61, Ala62, Ser113, Gly122, Leu143, Val145, Arg157	Val115, Asp121, His144, Thr146, Lys149, Thr153
	1,3,12-Nonadecatriene	-5	Tyr60, Val61, Ala62, Leu143, Val145, Lys147, Lys149	Ala111, His112, Ser113, His144, Thr146, Thr153, Arg157
	Ethyl stearate	-4.3	Val61, Ser113, Leu143, Val145, Lys149, Arg157	Tyr60, Ala62, Val115, Asp121, Gly122, His144, Thr146, Thr153
	Glycidyl palmitate	-4.7	Arg59, Val61, Pro65, Val115, Asp121, Gly122, Leu143	Tyr60, Gly64, Ser113, Gly116, Cys119, Glu120, Arg157
	Ethyl 9,10-epoxyoctadecanoate	-5.2	Arg59, Val61, Val115, Leu143, Val145, Lys149, Thr153, Arg157	Gly64, Pro65, His67, Gly68, Ala111, His112, Ser113, Asp121, Gly141, Ile142
	9-Octadecenoic acid, ethyl ester	-5.1	Phe56, Arg59, Val61, Val115, Leu143, Lys149, Thr153	Gly64, Pro65, His67, Gly68, Ser113, Gly141, Ile142, His144, Val145, Thr146, Arg157
	Diisobutyl adipate	-4.9	Val61, Ala62, Thr146, Arg157	Tyr60, Ala111, His112, Ser113, Leu143, His144, Val145, Lys149, Thr153
	Ethyl palmitate	-4.7	Arg59, Val61, Pro65, Val115, Leu143	Tyr60, Gly64, His67, Gly68, Gly69, Gly116, Asn139, Leu140 Gly141, Ile142

	2,4-Decadienal	-4.7	Arg59, Val61, Pro65, His67, Gly68, Val115, Leu143	Gly64, Gly141
	3,6-Diazahomoadamantan-9-ol	-4.4	Ser249	Ser243, Lys244, Asn250, Asp274, Lys275, Phe310
	2-Oxatricyclo[4.3.1.0(3,8)]decane	-4.1	Val61, Pro65, Val115, Leu143	Gly64
NFE2L2 (7X5E)	Stigmasterol	-6.7	Phe481, Arg499, Arg502, Arg503, Lys506	Asn482, Met485, Asn507, Ala510
	Cycloartenol	-7.4	Lys506	Val478, Arg499, Arg502, Arg503
	Clionasterol	-6.3	Val478, Arg499, Arg502, Arg503	Pro477, Phe481, Lys506
	Fucosterol	-6.2	Val478, Arg499, Arg502, Arg503	Phe 481, Asn482, Arg502, Lys506
	6,7-Dimethyltetralin-1,5,8-trione	-6.2	Arg502, Arg503, Lys506	Val478, Phe481, Arg499
	alpha-Selinene	-6.2	Phe481, Arg499, Arg502, Arg503	Lys506
	Glyceryl monooleate	-4.5	Phe481, Arg499, Arg502, Arg503, Lys506	Ala496, Asp500
	Butyl linoleate	-4.3	Val478, Phe281, Arg499, Arg502, Arg503	Ans482, Lys506
	Butanoic acid, [2-(1-hexenyl)cyclopropyl]methyl ester, [1R-[1.alpha.,2.alpha.(E)]]-	-4.4	Arg499, Arg502, Arg503	Val478, Phe481, Ala496, Lys506
	Glycidyl oleate	-4.7	Phe481, Arg499, Arg502, Arg503	Val478, Ala496, Lys506
	1,3,12-Nonadecatriene	-4.4	Val478, Phe481, Arg499, Arg502, Arg503, Lys506	Asn482
	Ethyl stearate	-4.2	Phe481, Ala496, Arg499, Arg502, Arg503	Lys506
	Glycidyl palmitate	-4	Phe481, Arg499, Arg502, Arg503, Lys506	Val478, Asn482
	Ethyl 9,10-epoxyoctadecanoate	-4.6	Phe481, Arg499, Lys506, Ala510	Val478, Asn507
	9-Octadecenoic acid, ethyl ester	-3.9	Phe481, ALa496, Arg499, Arg502, Arg503	Val478, Asn482
	Diisobutyl adipate	-4.9	Phe481, Ala496, Arg502, Arg503	Val478, Arg499, Asp500, Lys506
	Ethyl palmitate	-4.2	Phe481, Ala496, Arg499, Arg502, Arg503	Val478, Asp500, Lys506
	2,4-Decadienal	-4.2	Phe481, Arg499, Arg502, Arg503	
	3,6-Diazahomoadamantan-9-ol	-4.6	Arg503	Phe481, Arg499, Arg502, Lys506
	2-Oxatricyclo[4.3.1.0(3,8)]decane	-4.6	Arg499, Arg502, Arg503	Phe481, Lys506
TLR4 (3FXI)	Stigmasterol	-6.8	Ala291, Val316, Val338, Ser381, Arg382	Arg234, Arg289, Tyr292, Ser317, Glu336, Thr357, Thr359, Ser360, Asp379
	Cycloartenol	-7.0	Ala291, Val316, His179, Leu180	Asp181, Ser107, Asp109, Lys230, Thr232, Arg234, Val259, Phe263, Arg289, Tyr292, Ser317
	Clionasterol	-6.7	Ala291, Val316, Val338, Asp379, Ser381, Arg382	Arg234, Arg289, Tyr292, Ser317, Glu336, Thr357, Thr359, Ser360

Fucosterol	-5.7	Ala291, Val316	Arg234, Phe263, Arg264, Tyr292, Ser317, Asn339
6,7-Dimethyltetralin-1,5,8-trione	-5.7	Arg264, Tyr292, Thr319	Leu293, Asp294, Tyr296, Ser317, Val318, Lys362
alpha-Selinene	-5.6	Leu212	Pro214, Met215, Asn235, Asn236, Phe237, Asp238, Glu266, Gly267
Glyceryl monooleate	-4.0	His179, Asp181, Ser183, Asp209, Lys230	
Butyl linoleate	-4.3	Tyr296, Thr319, Lys362	Arg264, Tyr292, Leu293, Asp294, Tyr295, Ser317, Val318, Arg322, Lys341
Butanoic acid, [2-(1-hexenyl)cyclopropyl]methyl ester, [1R-[1.alpha.,2.alpha.(E)]]-	-4.0	Arg234, Phe263, Arg264, Ala291, Val316, Val338	Glu262, Arg289, Ser317
Glycidyl oleate	-3.9	His179, Lys230, Thr232	Glu154, Asn156, Leu180, Asp181, Ser207, Leu208, Asp209, Arg257
1,3,12-Nonadecatriene	-3.2	Leu212, Pro214	Asn235, Asn236, Phe237, Asp238, Glu266, Gly267
Ethyl stearate	-4.4	Lys230, Thr232, Arg234, Phe263, Arg264, Ala291, Tyr292, Val316	Asp209, Ser211, Val259, Arg289, Ser317
Glycidyl palmitate	-4	Val134, Ala158, His159, Ser211, Leu212, Arg234, Phe263	Asn156, Asp181, Ser183, Ser184, Asp209, Lys230, Thr232, Val259
Ethyl 9,10-epoxyoctadecanoate	-4.1	Lys230, Thr232, Arg234, Phe263, Arg264, Tyr292, Val316	Asp209, Ser211, Val259, Arg289, Ala291, Ser317
9-Octadecenoic acid, ethyl ester	-4.2	Tyr295, Tyr296, Thr319, Lys362	Tyr292, Leu293, Asp294, Asp298, Ser317, Val318, Arg322, Lys341
Diisobutyl adipate	-4.6	Arg234, Phe263, Arg264, Arg289, Ser317	Lys230, Thr232, Val259, Asn265, Ala291, Tyr292, Val316
Ethyl palmitate	-4.1	Val259, Phe263, Arg264, Arg289, Ala291, Tyr292, Val316	Lys230, Thr232, Arg234, Asn265, Ser317
2,4-Decadienal	-3.8	Phe263, Arg264, Ala291, Tyr292, Val316	Arg234, Arg289, Ser317
3,6-Diazahomoadamantan-9-ol	-4.6	Asn235, Phe237, Asp238	Leu212, Asn213, Pro214, Met215, Asn236, Gly267
2-Oxatricyclo[4.3.1.0(3,8)]decane	-4.5	Tyr292, Thr319	Leu293, Asp294, Tyr296, Ser317, Val318, Lys341, Lys362
